# The Chromosome-Scale Assembly of the *Curcuma alismatifolia* Genome Provides Insight Into Anthocyanin and Terpenoid Biosynthesis

**DOI:** 10.3389/fpls.2022.899588

**Published:** 2022-06-15

**Authors:** Qing Dong, Qing-Cheng Zou, Li-Hui Mao, Dan-Qing Tian, Wei Hu, Xue-Rui Cao, Hua-Qiao Ding

**Affiliations:** Zhejiang Institute of Landscape Plants and Flowers, Hangzhou, China

**Keywords:** anthocyanin, *C. alismatifolia*, evolution, genome, terpenoid

## Abstract

*Curcuma alismatifolia*, a bulbous flower known for its showy bracts, is widely used around the world as a cut flower, potted, and garden plant. Besides its ornamental value, this species is rich in terpenoid metabolites and could serve as a resource for essential oils. Here, we report a chromosome-level genome assembly of *C. alismatifolia* and describe its biosynthetic pathways for anthocyanins and terpenoids. This high-quality, assembled genome size is 991.3 Mb with a scaffold N50 value of 56.7 Mb. Evolutionary analysis of the genome suggests that *C. alismatifolia* diverged from *Zingiber officinale* about 9.7 million years ago, after it underwent a whole-genome duplication. Transcriptome analysis was performed on bracts at five developmental stages. Nine highly expressed genes were identified, encoding for six enzymes downstream of the anthocyanin biosynthetic pathway. Of these, one gene encoding F3′5′H might be a key node in the regulation of bract color formation. Co-expression network analysis showed that MYB, bHLH, NAC, and ERF transcription factors collectively regulated color formation in the bracts. Characterization of terpenoid biosynthesis genes revealed their dispersal and tandem duplications, both of which contributed greatly to the increase in the number of terpene synthase genes in *C. alismatifolia*, especially to species-specific expansion of sesquiterpene synthase genes. This work facilitates understanding of genetic basis of anthocyanin and terpenoid biosynthesis and could accelerate the selective breeding of *C. alismatifolia* varieties with higher ornamental and medicinal value.

## Introduction

*Curcuma alismatifolia* Gagnep. is an ornamental, bulbous flower belonging to the Zingiberaceae family. It originated from the tropical and subtropical areas of northern Thailand and Cambodia. During the past few decades, it has gained popularity in the international market, where it is widely used as a cut flower, potted, and garden plant in various countries, including China, Germany, the Netherlands, New Zealand, Japan, and the United States (Ruamrungsri, 2015).

The ornamental value of *C. alismatifolia* is based on its showy inflorescence, which is comprised of several large, verticillate bracts on a long peduncle. The basal bracts are green, each subtending a small axillary flower with a delicate purple labellum. The small flowers are almost invisible from a distance due to the shielding of the bracts. The prominent elliptical distal bracts are generally varying degrees of pink with green tips, which determine the attractiveness of the inflorescences. It has been reported that the color of the distal bracts is determined by the accumulation of anthocyanin pigments (Nakayama et al., 2000; Koshioka et al., 2015). Anthocyanins are a class of plant flavonoid secondary metabolites, which are helpful not only in attracting pollinators and facilitating seed dispersion, but also play key roles in biotic and abiotic stress responses (Saigo et al., 2020). The process of anthocyanin biosynthesis is comprised of a series of catalyzing enzymes whose expressions are mainly regulated by complexes formed by transcription factors R2R3-MYB, bHLH, and WD40 (Saigo et al., 2020). The molecular mechanisms of anthocyanin biosynthesis have been well characterized in several species; however, little information is currently available for the anthocyanin biosynthesis of *C. alismatifolia*.

The Zingiberaceae family is famous for its abundance of bioactive metabolites; many species in this family are widely used as traditional medicine or spices throughout the world (Barbosa et al., 2017). As a member of the Zingiberaceae, *C. alismatifolia* has been found to be rich in sesquiterpenes metabolites in its rhizome essential oils, such as xanthorrhizol, ar-curcumene, and β-curcumene (Theanphong and Mingvanish, 2017; Kochaphum et al., 2019). Terpene is the largest class of natural products in plants, with over 50,000 structures reported.^1^ Terpenes play numerous roles that are vital to basic plant processes, such as defense against pests and diseases and adaptation to environmental conditions. They also have a number of potential applications across the food, pharmaceutical, and agriculture industries. Xanthorrhizol, the most abundant sesquiterpene in *C. alismatifolia*, possesses several bioactive functions, such as anticancer, anti-inflammatory, antioxidant, and hepatoprotective effects (Oon et al., 2015). The essential oil of *C. alismatifolia* exhibited strong antioxidant activity, showing neuroprotective and neurogenic activity against P19-derived neurons at 1 ng/ml (Kochaphum et al., 2019). Generation of terpenes in plants is largely species-specific. Individual species, driven by selective pressure to adapt to their specific ecological niche, generally produce only a small fraction of plant terpenes because terpenes play crucial roles in mediating interactions with various ecological habitats (Chen et al., 2011; Karunanithi and Zerbe, 2019). Up to now, terpene biosynthetic pathways in Zingiberaceae family are largely unknown, including the biosynthetic pathways for *C. alismatifolia*.

During the past decade, traditional breeding programs have been launched in many countries for *C. alismatifolia* (Taheri et al., 2016; Ke et al., 2020). Interest has also been generated towards the genetic diversity, gene identification, tissue culture, biotic, and abiotic stress response for this specie (Ruamrungsri, 2015; Dey et al., 2019; Taheri et al., 2019; Li et al., 2021). Genome sequence is critical for efficient molecular breeding and genetic research of plant species. In this study, we built a chromosome-level genome for *C. alismatifolia* and deciphered the biosynthetic pathways of its anthocyanin and terpene metabolites. We expect that this genome could contribute immensely to the selective breeding of *C. alismatifolia* varieties with higher ornamental and medicinal values.

## Materials and Methods

### Genome Sequencing and Assembly

**Figure 1:**
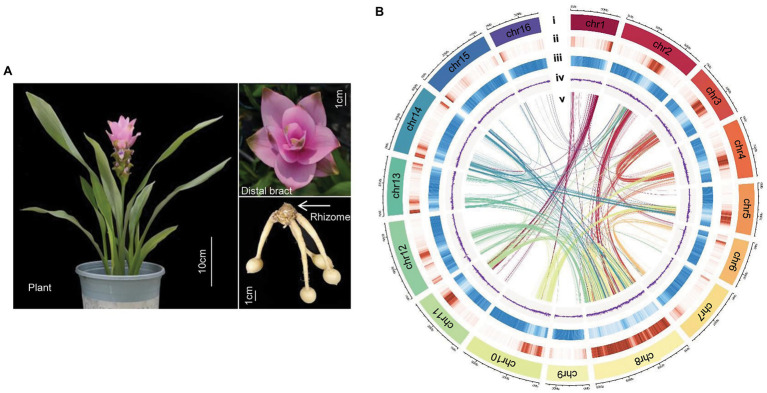
Genome of *Curcuma alismatifolia*. **(A)** Plant, distal bract and rhizome of *C. alismatifolia*. **(B)** Chromosomal features: (i) chromosome, (ii) gene density, (iii) repeat sequence density, (iv) GC (guanine- cytosine) content, and (v) syntenic blocks.

The most popular cultivar on the world market, Chiangmai Pink, was used for sequencing and assembly in this study (Figure 1A). *Curcuma alismatifolia* plants were grown in field in Hangzhou, Zhejiang Province in 2019. Fresh leaf, root, stem, distal bract, and flower were collected. Only leaf samples were used for DNA sequencing; all tissue types were used for transcriptome sequencing.

Genomic DNA was extracted from leaf samples using the CTAB extraction method and three libraries were constructed (Supplementary Table 1). A short length (~350 bp) library was constructed using the manufacturer’s procedures (Huang et al., 2018) and then sequenced using the DNBSEQ™ platform for genome surveying and base correction after assembly. A 20-kb SMRTbell library was constructed using PacBio preparation protocols (Travers et al., 2010) and then sequenced using the PacBio Sequel I platform for full genome assembly. A Hi-C library was constructed as previously described (Van Berkum et al., 2010) and sequenced using the DNBSEQ™ platform for chromosome level genome assembly. Total RNA was extracted from leaf, root, stem, bract and flower using the RNeasy plant Mini Kit (QIAGEN, Germany). Transcriptome analysis was performed on these tissues using DNBSEQ™ for gene prediction.

The genome survey was performed through the k-mer method using DNBSEQ™ reads. The 17-mer frequency distribution was analyzed using Jellyfish v2.1.4 (Marçais and Kingsford, 2011), and then genome size, heterozygosity, and repeat sequence were estimated using GenomeScope v1.0 (Vurture et al., 2017). *De novo* assembly of the genome was performed based on the PacBio long reads using Falcon v1.2.4 (Chin et al., 2016). The DNBSEQ™ short-read data was aligned to the contigs for correction using BWA v0.7.15 (Li and Durbin, 2009) and Pilon v1.22 (Walker et al., 2014). Redundancy in contigs was eliminated using HaploMerger2 (Huang et al., 2017). For chromosome level scaffolding, the Hi-C reads were aligned against the draft genome using JUICER v1.5.6 (Durand et al., 2016), and the data was filtered and evaluated using HiC-Pro v2.5.0 (Servant et al., 2015). The valid Hi-C reads pairs were applied to cluster, order, and orient the assembled contigs at a chromosome-level using JUICER v1.5.6 (Durand et al., 2016) and 3D-DNA v180922 (Dudchenko et al., 2017). Completeness and accuracy of the assembled genome was evaluated using BUSCO v3 with the embryophyta_odb10 database (Simão et al., 2015).

### Annotation of Repetitive Elements

Transposon elements in the *C. alismatifolia* genome were annotated by homology search against known repeat databases and *de novo* prediction. For the homology search, RepeatMasker v4.0.7^2^ and RepeatProteinMasker v4.0.7 (Tarailo-Graovac and Chen, 2009) softwares were used to search the repeat elements in the genome based on Repbase library v21.12.^3^ For *de novo* prediction, a repeat library was established based on LTR_FINDER v1.07 (Xu and Wang, 2007) and RepeatModeler v5.8.8 (see Footnote 2), and the repeat elements were predicted using RepeatMasker. In addition, tandem repeats in *C. alismatifolia* genome were detected using Tandem Repeats Finder v4.09 (Benson, 1999).

### Gene Prediction and Functional Annotation

Homology- and transcriptome-based approaches were combined to predict genes in the *C. alismatifolia* genome. For homology-based prediction, protein sequences from *M. acuminata*, *O. sativa*, *Sorghum bicolor*, and *Zea mays* were aligned to the assembled genome of *C. alismatifolia* using Exonerate v2.2.0 (Slater and Birney, 2005). For transcriptome-based annotation, the RNA-seq reads of different tissues were *de novo* assembled using Trinity v2.0.6 (Grabherr et al., 2013), and the redundancies of the assembled transcripts were removed using TGICL v2.1 (Pertea et al., 2003). The refined gene models were obtained from the assembled transcripts using PASA.^4^ Gene models from the two approaches were merged using MAKER v2.31.11 (Holt and Yandell, 2011).

### Phylogenetic Analysis and Whole-Genome Duplication Analyses

The genome of *C. alismatifolia* was compared to the genome sequences of 12 other plants, including *Zingiber officinale*, *Musa acuminate*, *Musa schizocarpa*, *Ananas comosus*, *Cocos nucifera*, *Oryza sativa*, *Phalaenopsis equestris*, *Arabidopsis thaliana*, *Populus trichocarpa*, *Solanum lycopersicum*, *Vitis vinifera*, and *Amborella trichopoda*. Gene families within these species were identified using Orthofinder v2.3.12, then amino acid sequence alignment was performed using Blastp v2.6.0 with an E-value threshold of 1e^−5^ (Camacho et al., 2009; Emms and Kelly, 2019). A phylogenetic tree was constructed using RAxML v8.2.10 (Stamatakis, 2014), based on single-copy gene families. The divergence time between *C. alismatifolia* and the other 12 species was estimated using MCMCTree in the PAML v4.9 package (Yang, 2007). The calibrated timescales were obtained from TimeTree website^5^: *Arabidopsis*–*P. trichocarpa* (79–109 Mya), *S. lycopersicum*–*V. vinifera* (105–115 Mya), *A. comosus*–*O. sativa* (94–115 Mya), *A. trichopoda*–*S. lycopersicum* (148–173 Mya). Expansion and contraction gene families were analyzed using CAFÉ v3.1 (Han et al., 2013). Whole-genome duplication (WGD) events were identified based on the synonymous substitutions (*Ks*) distribution, which was calculated using PAML v4.9 (Yang, 2007). The *Ks* values of *C. alismatifolia*–*Z. officinale* orthologs with the speciation dating of the two species allowed the calculation of the number of substitutions per synonymous site per year [divergence date = *Ks*/(2r)]. The same r value and the *Ks* value of *C. alismatifolia*–*C. alismatifolia* orthologs were used to calculate WGD ages. Gene ontology (GO) and KEGG enrichment analysis was conducted using the R package ClusterProfiler (Wu et al., 2021). The *q* value <0.05 was considered significant.

### Identification of Genes Involved in Anthocyanin and Terpenoid Biosynthesis

Fifty-two *Arabidopsis* genes, including 21 anthocyanin biosynthesis genes and 31 terpenoid backbone biosynthesis genes (Supplementary Table 2), were used as query sequences to identify their homologs in eight monocot species, including *C. alismatifolia*, *Z. officinale*, *M. acuminate*, *M. schizocarpa*, *A. comosus*, *C. nucifera*, *O. sativa*, and *P. equestris*. *Arabidopsis* protein sequences were downloaded from the KEGG^6^ or TAIR^7^ database. A BLASTP search was performed using Diamond with an E-value threshold of 10^−10^ (Buchfink et al., 2014). The identified genes were further confirmed with KEGG and Pfam annotations. Genes containing both pfam03936 and pfam01397 domains were identified as TPS genes. Sequence alignment was performed for terpene synthase proteins using Muscle v3.8 (Edgar, 2004). The phylogenetic tree was constructed by Fasttree v2 using Maximum Likelihood method (Price et al., 2010). The duplicate type of TPS genes was determined using duplicate gene_classifier_program integrated in the MCSanX package (Wang et al., 2012). The synteny blocks between *C. alismatifolia* and *Z. officinale* were identified using MCScan python version.^8^

### Transcriptome Analysis and Real-Time PCR

Distal bracts were collected from the five developmental stages, which were used to analyze genetic basis of color formation of bracts. Rhizome, root, leaf, bract and flower were collected at flowering stage, which were used to investigate expression of terpenoid biosynthesis genes. Transcriptome analysis was performed using the Illumina Novaseq 6000, with three replicates. The clean reads were mapped on the genome using HISAT2 v2.1.0 (Kim et al., 2015) and gene sequences using Bowtie2 v2.2.5 (Langmead, 2010). Gene expression value (FPKM) was calculated using RSEM v1.2.8 (Li and Dewey, 2011). Differential gene expression (DEG) among different stages was analyzed using DEseq2 v3.6.1 (Love et al., 2014).

Transcription factors in *C. alismatifolia* were identified using PlantRegMap (Tian et al., 2019). Co-expression networks between the anthocyanin biosynthetic genes and transcription factors were constructed based on DEGs according to the method of Chang et al. (2019). Co-expression network analysis (WGCNA) was performed based on all genes detected by transcriptome sequencing using a web-tool.^9^ The *cis*-regulatory elements in the promoter regions of anthocyanin biosynthetic genes using the PlantCARE database.^10^

Real-time PCR was used to validate the results of transcriptome analysis of anthocyanin biosynthetsis. The RNA of distal bracts from the five stages was used to synthesized the 1st cDNA using ReverTra Ace™ qPCR Master Mix (TOYOBO, Japan). Real-time PCR was conducted on Applied Biosystems StepOnePlus Real-Time PCR System using SYBR qPCR Mix Kit (TOYOBO, Japan), with three biological replicates and three technical replicates. *Actin1* was used as an endogenous control. Analysis was performed according to the 2^−∆∆Ct^ method. The primers were designed using Primer 5.0 and listed in Supplementary Table 2.

## Results

### Genome Sequencing and Assembly

We used a combined strategy of DNBSEQ™, PacBio, and Hi-C technologies to assemble the *C. alismatifolia* genome. A total of 123.3 Gb of clean, short reads was generated by the DNBSEQ™ sequencing system (Supplementary Table 1). A k-mer analysis was performed based on these data, and the genome size was estimated to be 1,096.6 Mb, with heterozygosity rate, repeats contents, and guanine-cytosine content (GC-content) of 1.42%, 79.28%, and ~35%, respectively (Supplementary Figure 1). A total of 139.7 Gb PacBio long reads with a contig N50 length of 30.2 kb was generated, which was ~128-fold coverage of the estimated genome size (Supplementary Table 1). The genome of *C. alismatifolia* was assembled based on the PacBio reads and corrected with DNBSEQ™ reads. The primary assembly was 989.4 Mb in length with a GC-content of 39.3%, which was consistent with the results of the genome survey.

In order to anchor the scaffolds to the chromosomes, a Hi-C library was constructed, and 131.1 Gb clean data was obtained (Supplementary Table 1). A total of 436.9 million paired-end reads were generated from the Hi-C data, of which 66.8 million read pairs provided valid interaction information for the chromosome assembly (Supplementary Figure 2). The final assembly was 991.3 Mb in length, with contig and scaffold N50 values of 0.45 and 56.67 Mb, respectively (Table 1). A total length of 943.4 Mb of the genomic sequence was anchored to 16 chromosomes (Figure 1B), accounting for 95.2% of the entire genomic sequence (Supplementary Table 3). BUSCO assessment identified 1,518 (94.0%) of the 1,614 highly conserved core proteins in the Embryophyta database, indicating the *C. alismatifolia* genome assembly was near-complete (Supplementary Table 4).

**Table 1:** Genome assembly and annotation statistics of *C. alismatifolia*.

**Chromosome-level genome assembly**	
Length genome (bp)	991,265,922
Number of contigs	6,431
Contig N50 length (bp)	450,000
Number of scaffolds	2,659
Scaffold N50 length (bp)	56,677,773
Mapping rate (%)	95.17
Total gap length (bp)	1,886,000
**Transposable elements (% of genome)**	
DNA length	24,346,733 (2.46)
LINE length	12,349,873 (1.24)
SINE length	232,521 (0.02)
LTR length	677,251,449 (68.45)
Other length	895 (0.00)
Unknown length	1,412,848 (0.14)
Total length	702,543,980 (71.01)
**Protein-coding genes**	
Predicted genes number	33,902
Average mRNA length (bp)	4,122.44
Average CDS length (bp)	1,267.65
Average exons per gene	5.76
Average exon length (bp)	220.02
Average intron length (bp)	598.46
Functionally annotated	33,625
**Noncoding protein genes**	
Number of miRNA	158
Average miRNA length (bp)	119.52
Number of tRNA	1,031
Average tRNA length (bp)	74.85
Number of rRNA	452
Average rRNA length (bp)	266.00
Number of snRNA	6,520
Average snRNA length (bp)	106.54

### Repeat and Gene Annotations

Both the *de novo* prediction and homology search against known repeat databases were used to annotate transposon elements in the *C. alismatifolia* genome. Overall, 702.5 Mb transposable elements were identified, which accounted for 71.0% of the genome (Table 1). Among them, long terminal repeat retrotransposons were the most abundant, accounting for 68.5% of the genome. In contrast, DNA transposons, long interspersed nuclear elements, and short interspersed nuclear elements had very low proportions, ranging from 0.02% to 2.46% of the genome.

A combined approach involving transcriptome and homology-based searches was adopted to predict genes encoding for proteins. In total, 33,902 protein-coding genes were annotated (Table 1). The mean lengths of genes, exons, and introns were 4,122.4, 220.0, and 598.5 bp, respectively. Among them, 33,625 genes (99.2%) were identified by at least one of five public databases, i.e., Non-redundant protein, SwissProt, TrEMBL, KEGG, and IntePro (Supplementary Figure 3). In addition, 8,161 noncoding RNAs were predicted, comprising of 158 miRNAs, 1,031 tRNAs, 452 rRNAs, and 6,520 snRNAs (Table 1; Supplementary Table 5).

### Phylogenetic Relationships and Whole-Genome Duplication Analyses

We compared our *C. alismatifolia* assembly with the genomes of 12 other plants. These plants included another Zingiberaceae species (*Z. officinale*), two species from the Musaceae family in the Zingiberales order (*M. acuminate* and *M. schizocarpa*), four other monocot species (*A. comosus*, *C. nucifera*, *O. sativa*, and *P. equestris*), four dicots (*Arabidopsis*, *P. trichocarpa*, *S. lycopersicum*, and *V. vinifera*), and the most basal extant flowering plant (*A. trichopoda*) as the outgroup.

**Figure 2:**
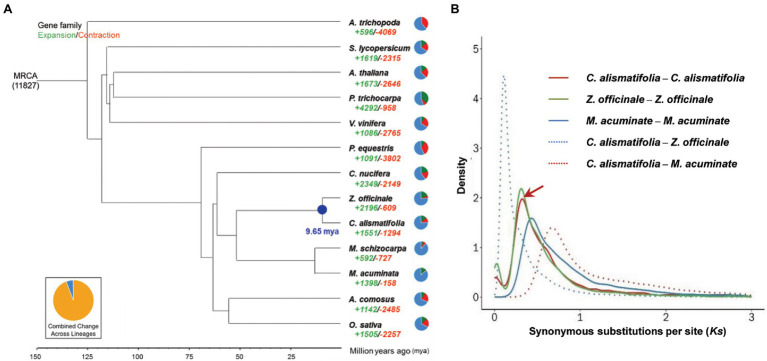
Evolution of *C. alismatifolia*. **(A)** Phylogenetic tree showing the evolution of gene families and divergence times. The green and red numbers represent the numbers of expanded and contracted gene families, respectively. In the pie charts, green, red, and blue portions show expanded, contracted, and constant gene families, respectively. Numbers at nodes represent divergence times (million years ago). **(B)** Distribution of synonymous substitutions per synonymous site (*Ks*). Peaks of intra-species *Ks* distributions indicate ancient whole-genome duplication events, and peaks of inter-species *Ks* distributions indicate divergence events. The arrow shows the recent whole genome duplication peak in *C. alismatifolia.*

A total of 16,419 gene families were found for *C. alismatifolia*, of which 13,018 gene families were shared by all 13 species surveyed. Three thousand four hundred one families were specific to *C. alismatifolia* (Supplementary Figure 4). Single-copy gene families among the 13 species were selected to construct phylogenetic trees. As expected, *C. alismatifolia* clustered with another Zingiberaceae species, *Z. officinale*, and these two species were most closely related to the Musaceae family species *M. acuminate* and *M. schizocarpa* (Figure 2A). Divergence dating analysis revealed that *C. alismatifolia* and *Z. officinale* diverged from each other approximately 9.7 million years ago (mya), and they both diverged from *M. acuminate* and *M. schizocarpa* around 51.4 mya. Distributions of *Ks* within genes in syntenic blocks were further analyzed. The results showed that *C. alismatifolia* underwent an WGD before it diverged from *Z. officinale* (Figure 2B). The date of WGD event of *C. alismatifolia* was estimated to be approximately 22.1 mya, which is similar to the 27 mya reported for *Z. officinale* (Cheng et al., 2021).

Further analysis revealed that 1,551 and 1,294 gene families were expanded and contracted in *C. alismatifolia*, respectively (Figure 2A). The expanded gene families were significantly enriched in 163 GO and 19 KEGG terms (*q* value <0.05; Supplementary Tables 6, 7). Many of these genes were assigned to chitin binding, chitin catabolic processes, and chitinase activity, implying that *C. alismatifolia* possesses a strong defense system against fungal pathogens (Gong et al., 2020). In addition, genes involved in gingerol biosynthesis were also expanded in *C. alismatifolia*, which is consistent with the abundance of gingerol in other members of the Zingiberaceae family (Sharifi-Rad et al., 2017). The contracted gene families were significantly enriched in 81 GO and 12 KEGG terms, which are mainly involved in UDP-glycosyltransferase activity, tropane and pyridine alkaloid biosynthesis, and plant hormone signal transduction (Supplementary Tables 8, 9).

### Exploration of Structural Genes and Their Regulators in Anthocyanin Biosynthesis Pathway

**Figure 3:**
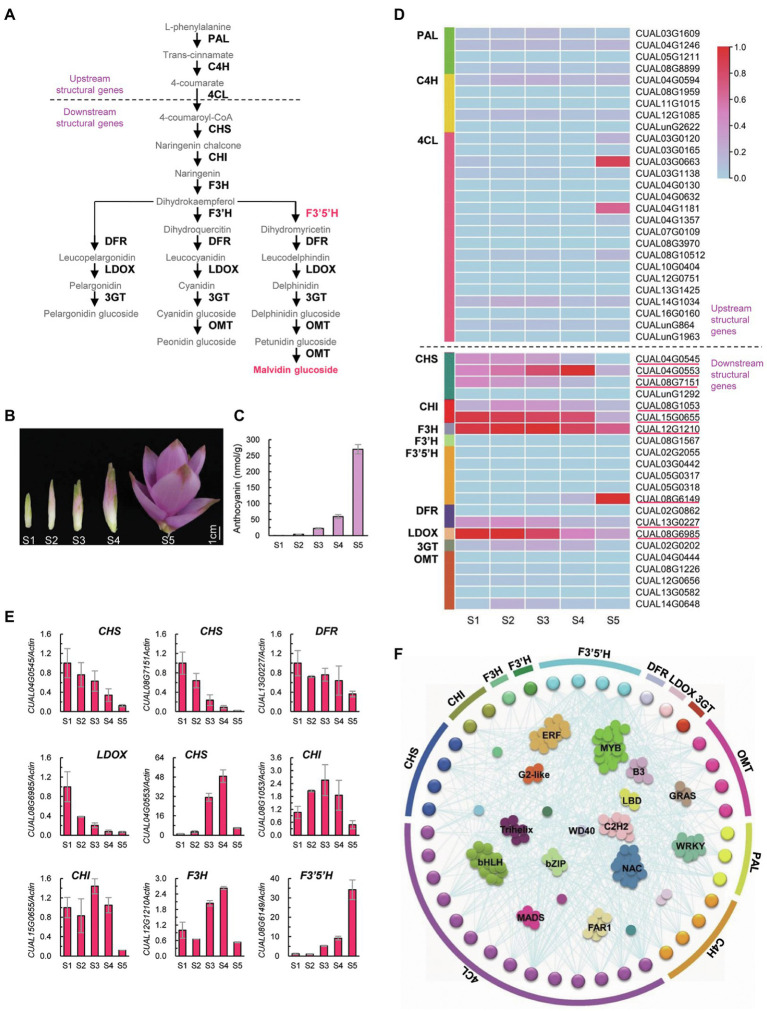
Biosynthesis of anthocyanin in *C. alismatifolia*. **(A)** Biosynthesis pathways of anthocyanin. Structural genes in anthocyanin biosynthetic pathway were divided into two groups, designated as “upstream structural genes” and “downstream structural genes” respectively. The upstream structural genes are implicated in the biosynthesis of precursors of flavonoids, while the downstream structural genes are specifically involved in anthocyanin biosynthesis. Malvidin, the major anthocyanin in bract in *C. alismatifolia*, is marked in red. PAL, phenylalanine ammonia lyase; C4H, cinnamate 4-hydroxylase; 4CL, 4-coumarate CoA ligase; CHS, chalcone synthase; CHI, chalcone isomerase; F3H, flavanone 3-hydroxylase; F3′H, flavonoid 3′-hydroxylase; F3′5′H, flavonoid 3′,5′ hydroxylase; DRF, dihydroflavonol 4-reductase; LDOX, leucoanthocyanidin dioxygenase; 3GT, UDP-glucose flavonoid 3-glucosyltransferase; and OMT, O-methyltransferases. **(B)** Distal bracts from five developmental stages. From left to right, youngest to oldest (S1–S5). **(C)** Anthocyanin contents in the distal bracts collected from the five stages. **(D)** Expression heatmap of the 49 structural genes at different stages of bract development. The expression values at the column scale were z-score normalized. Low to high expression is indicated by a change in color from blue to red. The nine highly expressed downstream genes are marked with red lines. **(E)** Real-time PCR analysis of the nine downstream structural genes with high transcription levels. The expression levels were normalized to *Actin1* (*CUAL02G1340*) and related to stage 1. Data are presented in mean ± SD (*n* = 3). **(F)** The co-expression network of the structural genes with transcription factors.

Anthocyanins are an important type of flavonoid, whose biosynthesis involves a series of catalyzing enzymes (Figure 3A). Upstream enzymes, including PAL, C4H, and 4CL, are linked in the biosynthesis of precursors of all flavonoids. Downstream enzymes, such as CHS, CHI, F3H, F3′H, F3′5′H, DFR, LDOX, 3GT, and OMT, are specifically involved in the biosynthesis of different types of anthocyanins (Pelletier et al., 1997). Twenty-one *Arabidopsis* structural genes encoding these 12 catalyzing enzymes were used as queries to identify homologs in *C. alismatifolia*. A total of 49 structural genes were identified, of which 18 genes were found to encode 4CL. For each of the remaining enzymes, one to five genes were detected.

Distal bracts of *C. alismatifolia* were collected from five developmental stages (Figure 3B) and used for anthocyanin content measurements and transcriptome analysis. The anthocyanin concentration in these bracts gradually increased with development (Figure 3C), which coincides with their color change. Most of the upstream structural genes showed a moderate or low expression during all five developmental stages (Figure 3D). This was consistent with their function: synthesizing precursors for all types of flavonoids, rather than specifically for anthocyanins. In contrast, for the downstream enzymes, at least one gene exhibited high expression levels at one of five development stages (Figure 3D). The transcription levels of these nine highly expressed downstream genes were further validated using real-time PCR (Figure 3E). Four of them, including *CUAL04G0545* and *CUAL08G7151* encoding CHS, *CUAL13G0227* encoding DFR, and *CUAL08G6985* encoding LDOX, were high expressed at developmental stage 1, and gradually down-regulated with bract development (Figure 3E). Four other genes, including *CUAL04G0553* encoding CHS, *CUAL08G1053* and *CUAL15G0655* encoding CHI, and *CUAL12G1210* encoding *F3H*, showed a fluctuating expression pattern, with their highest expression observed at stage 3 or 4 but sharply downregulated at stage 5. The remaining gene, *CUAL08G6149* encoding F3′5′H, was gradually up regulated with the development of bracts, which coincides with their anthocyanin accumulation.

Expression of structural genes is tightly associated with anthocyanin accumulation, which is mainly regulated by transcription factors (Saigo et al., 2020). We thus annotated transcription factors in *C. alismatifolia*, and then mapped a gene regulation network between the structural genes and transcription factors. Forty structural genes were found to be tightly correlated with 389 transcription factors in terms of expression pattern (Figure 3F). Among these transcription factors, *MYB* family members were most numerous, followed by *bHLH*, *NAC*, and *ERF* family members. We also conducted a WGCNA with all genes detected by transcriptome sequencing. A total of 15 WGCNA modules were identified (Supplementary Figure 5), of which the module “pink” was found to be highly correlated with anthocyanin accumulation (Supplementary Figure 6). This module was the largest among the 14 modules, containing 12 structural genes and 238 transcription factors (Supplementary Table 10). These genes were significantly enriched in 14 KEGG terms, one of which was “flavonoid biosynthesis” (Supplementary Table 11).

### Determination of Functional Genes in Terpenoid Biosynthesis Pathway

**Figure 4:**
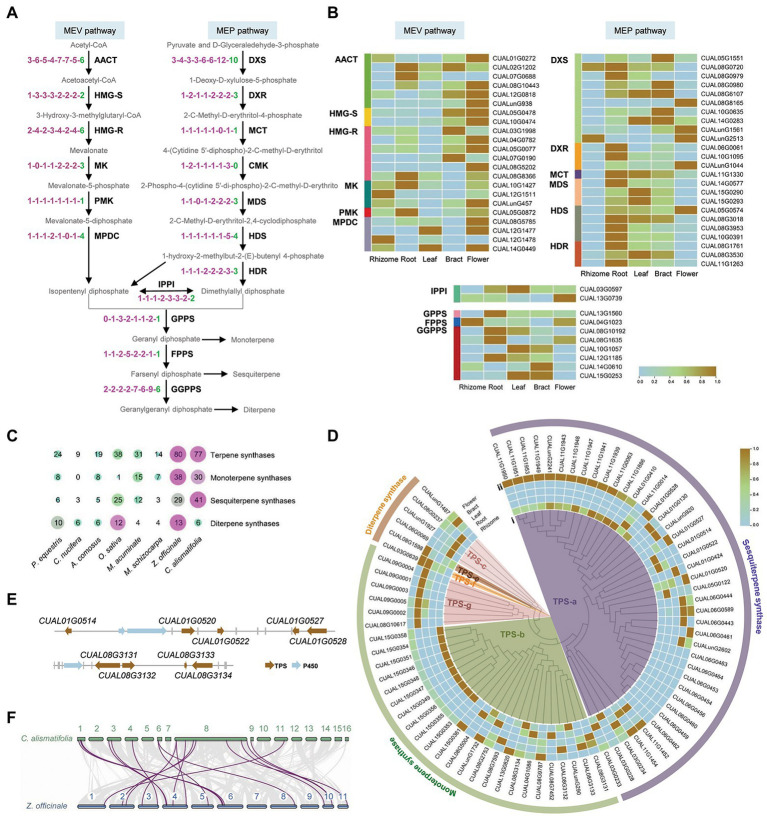
Biosynthesis of terpenoids in *C. alismatifolia*. **(A)** The backbone biosynthetic process of terpenoid. AACT, acetyl-CoA C-acetyltransferase; HMG-R, hydroxymethylglutaryl-CoA reductase; HMG-S, hydroxymethylglutaryl-CoA synthase; MK, mevalonate kinase; PMK, phosphor-mevalonate kinase; MPDC, diphosphomevalonate decarboxylase; DXS, 1-deoxy-D-xylulose-5-phosphate synthase; DXR, 1-deoxy-D-xylulose-5-phosphate reductoisomerase; MCT, 2-C-methyl-D-erythritol 4-phosphate cytidylyltransferase; CMK, 4-(cytidine-5-diphospho)-2-C-methyl-D-erythritol kinase; MDS, 2-C-methyl-D-erythritol 2,4-cyclodiphosphate synthase; HDS, (E)-4-hydroxy-3-methylbut-2-enyl-diphosphate synthase; HDR, 4-hydroxy-3-methylbut-2-enyl-diphosphate reductase; IPPI, isopentenyl-diphosphate △-isomerase; GPPS, dimethylallyltranstransferase/geranyl diphosphate synthase; FPPS, (2E,6E) farnesyl diphosphate synthase; and GGPPS, geranylgeranyl diphosphate synthase. Number of gene encoding these enzymes was compared among eight monocotyledonous plants, i.e., *P. equestris*, *Cocos nucifera*, *A. comosus*, *O. sativa*, *M. acuminate*, *M. schizocarpa*, *Zingiber officinale*, and *C. alismatifolia*. The notation “1-1-1-1-1-1-1” indicates one homologous gene was identified among each plant in the order listed in the previous sentence. **(B)** Expression heatmap of the 56 terpenoid backbone biosynthetic genes in the five tissues. The expression values at the row scale were z-score normalized. Low to high expression is indicated by a change in color from blue to brown. **(C)** The gene number of terpene synthases (TPS) in the eight plants. **(D)** The phylogenetic tree and expression heatmap of terpene synthase genes in *C. alismatifolia*. (i) phylogenetic tree and (ii) expression heatmap. **(E)** Gene clusters of TPS and P450 genes in *C. alismatifolia*. **(F)** Colinear analysis of TPS genes in *C. alismatifolia* (top) and *Z. officinale* (bottom).

Terpenes are biosynthesized from the isoprenoid precursors diphosphate and dimethylallyl diphosphate, which derive from the mevalonate (MEV) and methylerythritol phosphate (MEP) pathways in plants. The isoprenoid precursors are further synthesized into a range of terpene backbones, including geranyl diphosphate, farnesyl diphosphate, and geranylgeranyl diphosphate. Thirty-one *Arabidopsis* genes, encoding 17 enzymes involved in this backbone biosynthetic process, were used as queries to identify homologs in *C. alismatifolia* and seven other monocotyledonous plants. A total of 56 genes were found in *C. alismatifolia*, second only to the 57 in another Zingiberaceae specie, *Z. officinale*. For eight of the 17 enzymes, *C. alismatifolia* had more gene copies than the species belonging to families other than Zingiberaceae (Figure 4A). Transcriptome analysis was performed for five tissues, including rhizome, root, leaf, bract and flower. The results revealed that most genes in the MVA pathway had higher expression in flower than other four tissues, while more genes in MEP pathway had higher expression in root than in the other tissues (Figure 4B).

Terpene synthases (TPS) play vital roles in terpenoid biosynthesis, which convert terpene backbones into structurally distinct terpenoid groups. We further annotated TPS genes in these eight species. Consistent with the copy number of terpenoid backbone biosynthesis genes, the two Zingiberaceae species had more TPS genes than other species. The numbers of TPS genes in *C. alismatifolia* and *Z. officinale* were 77 and 80, respectively, while those in the other six species only ranged from nine to 38 (Figure 4C). The 77 *C. alismatifolia* TPS genes were unevenly distributed on 10 chromosomes. Seven gene clusters were observed, located on chromosome 1, 3, 6, 8, 9, 11, and 15, respectively (Supplementary Figure 7). Duplication modes were investigated for the *C. alismatifolia* TPS genes. Twenty-seven, 26, 17, and 7 of these genes could be related to dispersed, tandem, proximal, and whole-genome duplication type of genes (Supplementary Table 12). This suggested that both dispersed and tandem duplications played important roles in the expansion and evolution of TPS genes in *C. alismatifolia*.

A phylogenetic tree was constructed for the *C. alismatifolia* TPS genes. These genes were classified into six groups, based on the reported classification of *Arabidopsis* TPS genes (Figure 4D; Supplementary Figure 8). The TPS-a group contained the largest number of 41 genes, while TPS-e and TPS-f group only contained one gene, respectively. Functions of these genes were annotated using the Terzyme prediction server (Priya et al., 2018). All 41 genes belonging to the TPS-a group were predicted to encode sesquiterpene synthases. Among the remaining genes, 30 and 6 were predicted to encode monoterpene and diterpene synthases, respectively (Figure 4D).

Almost all the TPS genes tended to express in a tissue-specific pattern (Figure 4D). For monoterpene synthases, more genes had higher expression in rhizome and bract than in root, leaf and flower. For sesquiterpene synthases, more genes had higher expression in flower and rhizome than the other three tissues. In addition, members in the gene cluster exhibited similar expression pattern. For example, all the nine members in the cluster located at the distal end of chromosome 11, including *CUAL11G1939*, *CUAL11G1941*, *CUAL11G1943*, *CUAL11G1947*, *CUAL11G1948*, *CUAL11G1949*, *CUAL11G1950*, *CUAL11G1951*, and *CUAL11G1953*, mainly expressed in flower and rhizome. Similar phenomenon was also observed for the gene cluster located at chromosome 9 and 15, members of which mainly expressed in bract and rhizome, respectively.

It is known that biosynthetic pathways are frequently clustered together on chromosomes. Clustered, specialized metabolic pathway genes were identified in *C. alismatifolia* genome using the PlantiSMASH analytical pipeline (Kautsar et al., 2017). Two clusters involving in terpenes were found (Figure 4E). One was located in chromosome 1, which consisted of five genes encoding sesquiterpene synthases and two genes encoding cytochrome P450, the largest repository for enzymes responsible for modifying terpenes to diversify their functions (Bathe and Tissier, 2019). Another cluster was located in chromosome 8, which contained four genes encoding monoterpene synthases and one gene encoding cytochromes P450.

## Discussion

*Curcuma alismatifolia* is an promising flower that was introduced as an ornamental plant in early 1980s (Ruamrungsri, 2015). Within a short span of three decades, it has become well known in the world market due to its showy bracts. For a long time, *C. alismatifolia* was mainly regarded as an ornamental plant. It is only in the last few years that the richness of its terpenoid metabolites with medicinal value has been recognized (Theanphong and Mingvanish, 2017; Kochaphum et al., 2019). In this study, we described a chromosome-scale genome assembly for *C. alismatifolia* and preliminarily analysis of the genetic basis of its bract color formation and terpene biosynthesis. These results could accelerate the breeding of *C. alismatifolia* varieties with both higher ornamental and medicinal values.

The accumulation of anthocyanin in plant tissues are mainly determined by the expression intensity of the structural genes in a temporal and spatial way (Hichri et al., 2011; Jaakola, 2013). We examined transcription levels of the structural genes during development of distal bracts, and identified nine highly expressed genes in the downstream of anthocyanin biosynthetic pathway (Figure 3E). These genes might play important roles in color formation of bract in *C. alismatifolia*. Among these nine genes, four expressed highly at stage 1 and four other expressed highly at stage 3 or 4. All the eight genes were clearly downregulated at stage 5. This implied that they mainly functioned in early or middle stages of color formation of the bract. The remaining gene, *CUAL08G6149* encoding F3′5′H, was found to progressively up regulated with development of bracts (Figure 3E), which coincides well with the increase of anthocyanin content. The *CUAL08G6149* was also the only one of these nine genes that was aggregated into the “pink” module with the highest correlation to anthocyanin accumulation in the WGCNA analysis (Supplementary Table 10). It was reported that malvidin 3-rutinoside was the major anthocyanin type in distal bracts of *C. alismatifolia* (Nakayama et al., 2000; Koshioka et al., 2015). As illustrated in Figure 3A, F3′5′H is a specific catalyzing enzyme in the malvidin biosynthesis process. It catalyzes the hydroxylation of dihydrokaempferol to produce dihydromyricetin, which eventually generates malvidin-based anthocyanins. It could be inferred that F3′5′H was the rate-limiting enzyme in anthocyanin synthesis in the bract of *C. alismatifolia* and that *CUAL08G6149* was the key node gene in regulating its color formation.

Transcription factors also contribute to anthocyanin biosynthesis, as they regulate the expression of the catalyzing enzymes through binding to the promoters of the structural genes. The MYB, bHLH, and WD40 transcription factors were believed to play core roles in this regulation network (Saigo et al., 2020). Similar results were also observed in our co-expression network analysis (Figure 3F). Among the transcription factors tightly correlated to the structural genes, MYB and bHLH family members were most numerous. Moreover, we identified 49 structural genes carry MYB-binding sites in their promoters, and 26 of them carry bHLH-binding sites (Supplementary Figure 9). These MYB and bHLH transcription factors might be direct regulators for these anthocyanin structural genes. The MYB transcription factor is one of the largest gene families in plants, which contain a conserved MYB domain. According to the number of the conserved domain, the MYB family is classified into four subgroups, of which R2R3-MYB transcription factors are the major activator of anthocyanin biosynthesis (Saigo et al., 2020). In the present study, we identified seven R2R3-MYB genes in the co-expression network among the structural genes and transcription factors (Supplementary Figure 10). They were found to tightly correlated with the 14 structural genes in terms of expression pattern, including two *C4H*, four *4CL*, two *CHS*, one *F3′H*, three *F3′5′H*, one *DFR*, and one *OMT* (Supplementary Figure 11). Two of the seven R2R3-MYB genes, *CUAL08G2506* and *CUAL14G1448*, showed similar expression trends to the *F3′5′H* gene *CUAL08G6149*, which may be the rate-limiting enzyme for bract anthocyanin accumulation. We conducted a real-time PCR experiment and confirmed this similar expression pattern (Supplementary Figure 12). These two R2R3-MYB genes may play important roles in the regulatory network of distal bract color formation in *C. alismatifolia*. We further constructed phylogenetic trees for the R2R3-MYB and bHLH genes (Supplementary Figures 13, 14). Five R2R3-MYB genes and one bHLH gene in *C. alismatifolia* were grouped into the same cluster with the reported regulators of anthocyanin biosynthesis in *Arabidopsis*, such as *AtMYB123/TT2*, *AtMYB90*/*PAP2*, *bHLH42*/*TT8*, *EGL3*, and *GL3* (Lloyd et al., 2017). One of R2R3-MYB gene, *CUAL08G4902*, was found to be highly correlated with one of the 4CL genes in terms of expression pattern (*r* = 0.97; Supplementary Figure 15). These genes are good candidates for studying the regulatory mechanisms of *C. alismatifolia* anthocyanin biosynthesis. In addition to MYB and bHLH, many transcription factor families are responsible for biotic and/or abiotic stress, such as NAC and ERF. These two also showed strong associations with the expression of structural genes. It has been reported that the pink color of the *C. alismatifolia* bract was diminished under weak light environments (Lin et al., 2017). Another *C. alismatifolia* cultivar with white bracts, “Snowwhite,” could exhibit weak pink coloration in its bracts under some environmental conditions. These stress-related transcription factors could serve as targets to study the molecular mechanisms of color change in *C. alismatifolia* bracts in response to environmental stresses.

Terpenoid is one of largest classes of plant metabolites, which not only play numerous, vital roles in plant development, but also have a number of potential applications across pharmaceutical and agriculture industries. In this study, we found that *C. alismatifolia* and *Z. officinale* had more gene copies for many terpenoid backbone biosynthetic enzymes than other species (Figure 4A). The TPS family, which is the most critical determinants for terpene structural diversity, also obviously expanded in these two species (Figure 4C). This was consistent with the fact that the *Zingiberaceae* plants are famous for their richness in bioactive metabolites (Barbosa et al., 2017). It has been reported that the expansion of the TPS family mainly occurred after species diversity (Chen et al., 2011; Karunanithi and Zerbe, 2019). The species-specific expansion was also observed in our analysis. Although both *C. alismatifolia* and *Z. officinale* exhibited expansion in this family and carried a similar number of genes, the large-scale expansion occurred mainly for sesquiterpene synthases in *C. alismatifolia*, while in *Z. officinale* the expansion occurred mainly for monoterpene synthases (Figure 4C). Among the 77 TPS genes of *C. alismatifolia*, only 19 were found to be orthologs of *Z. officinale* (Figure 4F), although the two species are closely related (Figure 2A). Moreover, most of these collinear relationships were identified between scattered distributed genes and members of gene cluster, rather than between members gene clusters (Supplementary Figure 7). This implied the expansion of TPS in the two species came from duplication of different orthologs. Of 19 orthologs, six encoded sesquiterpene synthases, accounting for only 14.6% of the 41 sesquiterpene synthase genes in *C. alismatifolia* (Supplementary Figure 7). This implied that most sesquiterpene synthase genes in *C. alismatifolia* were generated after it diverged from *Z. officinale*. Most notable are the two sesquiterpene synthase gene clusters located on chromosomes 6 and 15 (Supplementary Figures 7, 16). The two clusters contained a total of 22 genes, accounting for 53.7% of all sesquiterpene synthase genes in *C. alismatifolia*. All these 22 genes were generated from tandem or proximal duplication (Supplementary Table 13), indicating these two duplication patterns played crucial roles in expansion of sesquiterpene synthase genes in *C. alismatifolia*. We further predicted the conserved motifs of the TPS proteins in the two species using the MEME program.^11^ The results showed that 41.5% (17/41) of sesquiterpene synthase in *C. alismatifolia* exhibited variations in motif organization (Supplementary Figure 17). For example, most members of the gene cluster on chromosome 6 lacked motif 6 and 8. In contrast, only 23.3% (7/29) of sesquiterpene synthase in *Z. officinale* showed variations in motif pattern. For monoterpene synthase, only 17.2% (5/29) *C. alismatifolia* members showed variations in motif organization. However, up to 47.4% (18/38) monoterpene synthase in *Z. officinale* exhibited variations, most of which lacked motif 10, 7, and 5. More variations were identified for *C. alismatifolia* sesquiterpene synthase genes and *Z. officinale* monoterpene synthase genes. This was consisted with the observation that the expansion occurred mainly for sesquiterpene synthases in *C. alismatifolia* while mainly for monoterpene synthases in *Z. officinale*. We also compared sequences in conserved RR(X)8W, DDXXD and RXR motifs between the two species. Similar to motif organization, more sequence variations were observed in sesquiterpene synthase in *C. alismatifolia* and monoterpene synthase in *Z. officinale* (Supplementary Figures 18, 19). For example, 17.1% (7/41) sesquiterpene synthases in *C. alismatifolia* and 36.8% (14/38) monoterpene synthases in *Z. officinale* showed sequence variations in RXR motif. In contrast, only 6.9% (2/29) sesquiterpene synthases in *Z. officinale* and 20.0% (6/30) monoterpene synthases in *C. alismatifolia* showed sequence variations in this motif. These variations in motif organization and conserved sequences might confer new functions to terpene synthases in the two species.

A total of 41 genes encoding sesquiterpene synthases were found in *C. alismatifolia*. This is the second largest sesquiterpene synthase subfamily reported in an angiosperm species to date, second in only to the 52 sesquiterpene synthase genes of *Eucalyptus* (Külheim et al., 2015). This is in accordance with the abundance of sesquiterpenes in *C. alismatifolia*. The chromosome-scale assembly of the *C. alismatifolia* genome lays a foundation for characterization of this large sesquiterpene synthases family. Xanthorrhizol is one of the most abundant sesquiterpene in *C. alismatifolia*, possessing a number of medical values (Oon et al., 2015). In the future, we will functionally characterize these sesquiterpene synthase genes by recombinant proteins expressed in *E. coli* to determine the genes involved in xanthorrhizol synthesis. In addition to *C. alismatifolia*, several other species in the genus *Curcuma* were found to be rich in xanthorrhizol, such as *Curcuma Xanthorrhiza* Roxb (Oon et al., 2015). The *C. alismatifolia* genome will also facilitate characterization of xanthorrhizol biosynthetic pathway in these species belonging to the same genus.

## Data Availability Statement

The datasets presented in this study can be found in online repositories. The names of the repository/repositories and accession number(s) can be found at: https://www.ncbi.nlm.nih.gov/, PRJNA734042.

## Author Contributions

QD designed the experiments and wrote original draft. QD, Q-CZ, L-HM, D-QT, WH, X-RC, and H-QD performed research and analyzed the data. All authors contributed to the article and approved the submitted version.

## Funding

This study was funded by the Youth Talent Program of Zhejiang Academy of Agricultural Sciences (2020R25R08E02), the Xiaoshan Science and Technology Plan Program (2020220), and the Key Research and Development Plan of Zhejiang Province (2019C02025).

## Conflict of Interest

The authors declare that the research was conducted in the absence of any commercial or financial relationships that could be construed as a potential conflict of interest.

## Publisher’s Note

All claims expressed in this article are solely those of the authors and do not necessarily represent those of their affiliated organizations, or those of the publisher, the editors and the reviewers. Any product that may be evaluated in this article, or claim that may be made by its manufacturer, is not guaranteed or endorsed by the publisher.

## Footnotes

^1^ https://dnp.chemnetbase.com/faces/chemical/ChemicalSearch.xhtml

^2^ http://www.repeatmasker.org

^3^ http://www.girinst.org/repbase

^4^ http://www.maplandia.com/ecuador/tungurahua/ambato/pasa

^5^ http://timetree.org/

^6^ https://www.kegg.jp

^7^ http://www.arabidopsis.org

^8^ https://github.com/tanghaibao/jcvi

^9^ http://www.biocloud.net/fxpt/app

^10^ http://bioinformatics.psb.ugent.be/webtools/plantcare/html

^11^ https://meme-suite.org/meme/tools/meme
