# Supplementary material for: The Chromosome-Scale Assembly of the Curcuma alismatifolia Genome Provides Insight Into Anthocyanin and Terpenoid Biosynthesis
Source: Front Plant Sci. 2022 Jun 15;13:899588. doi: 10.3389/fpls.2022.899588 (PMC9241516; doi:10.3389/fpls.2022.899588)
Supplement: Supplementary file 2 [file Data_Sheet_2.docx]

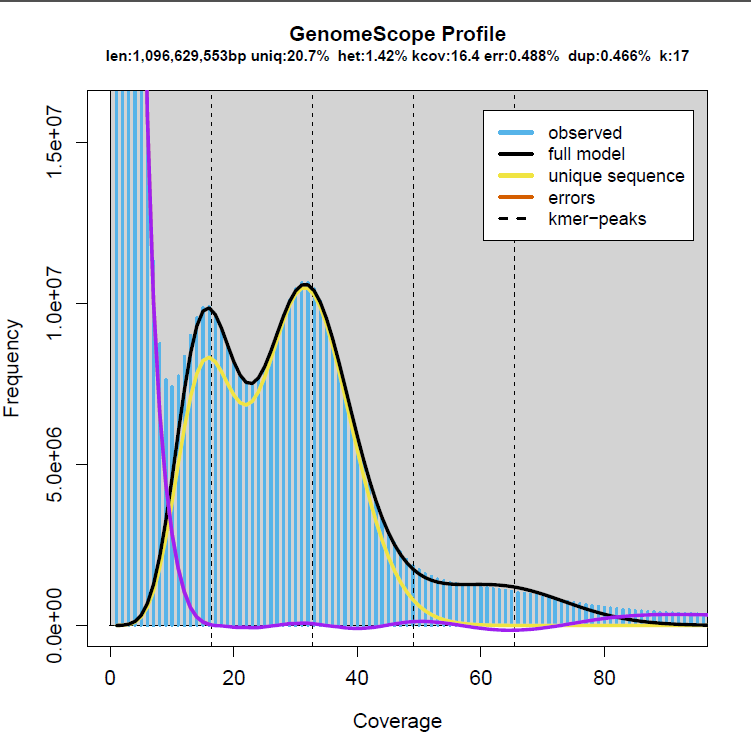


**Supplementary Figure 1.** Frequency distribution of the 17-mer graph analysis used for genome survey.

**
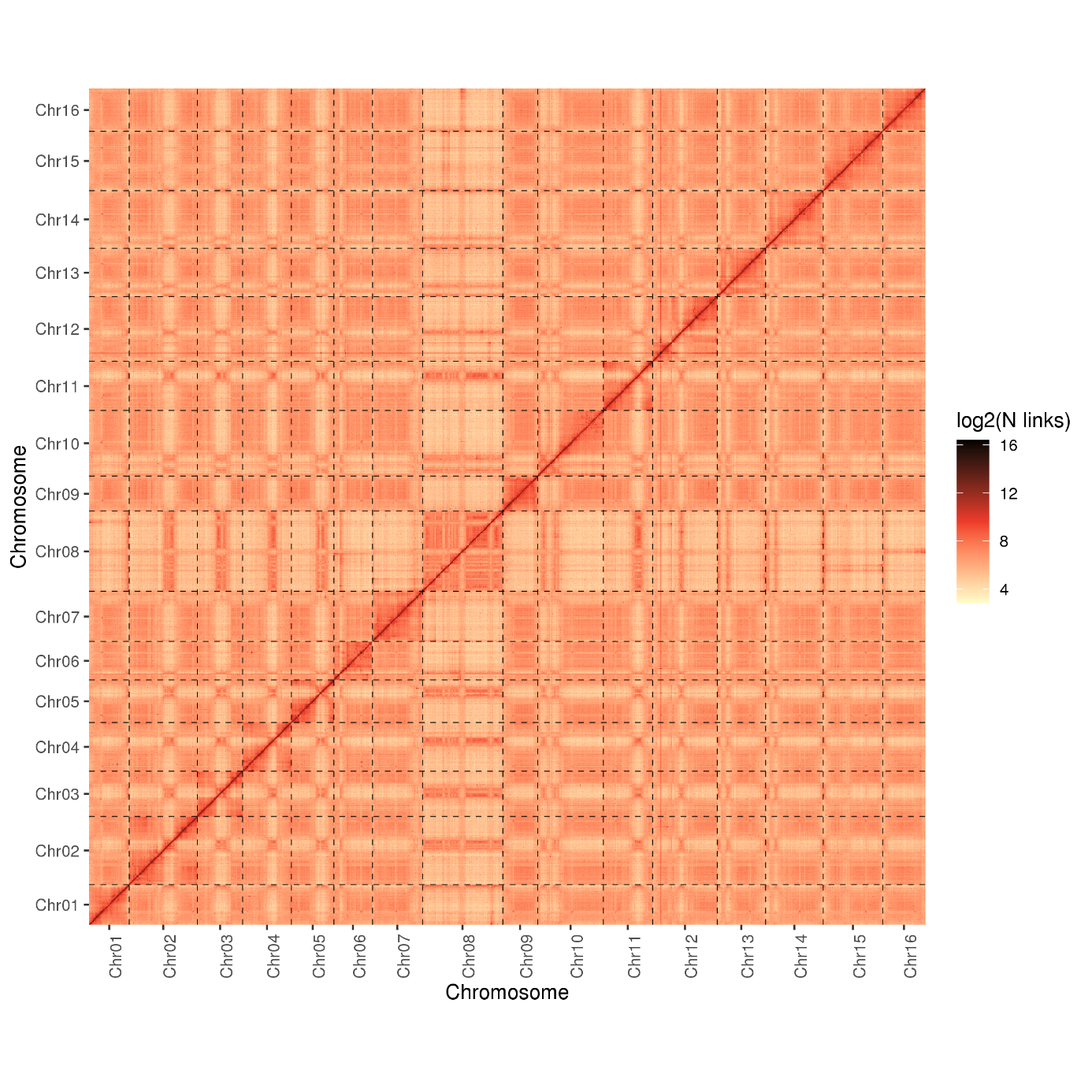
 Supplementary Figure 2.** Heatmap of Hi-C contact information of the 16 chromosomes.


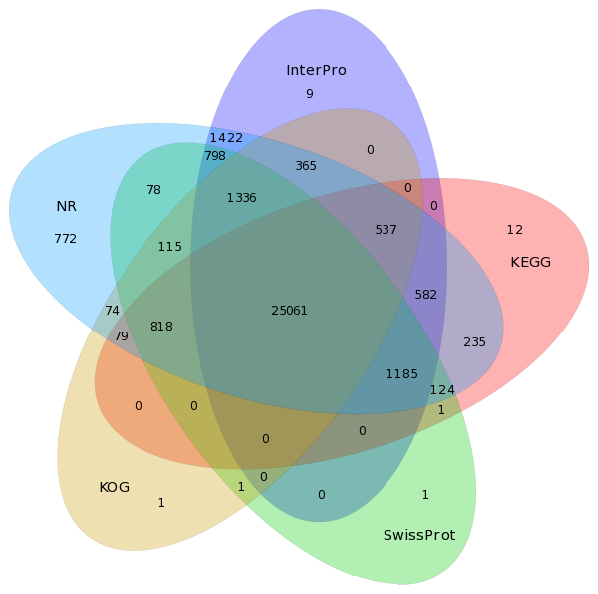


**Supplementary Figure 3.** Statistics of functional annotated protein-coding genes.

**
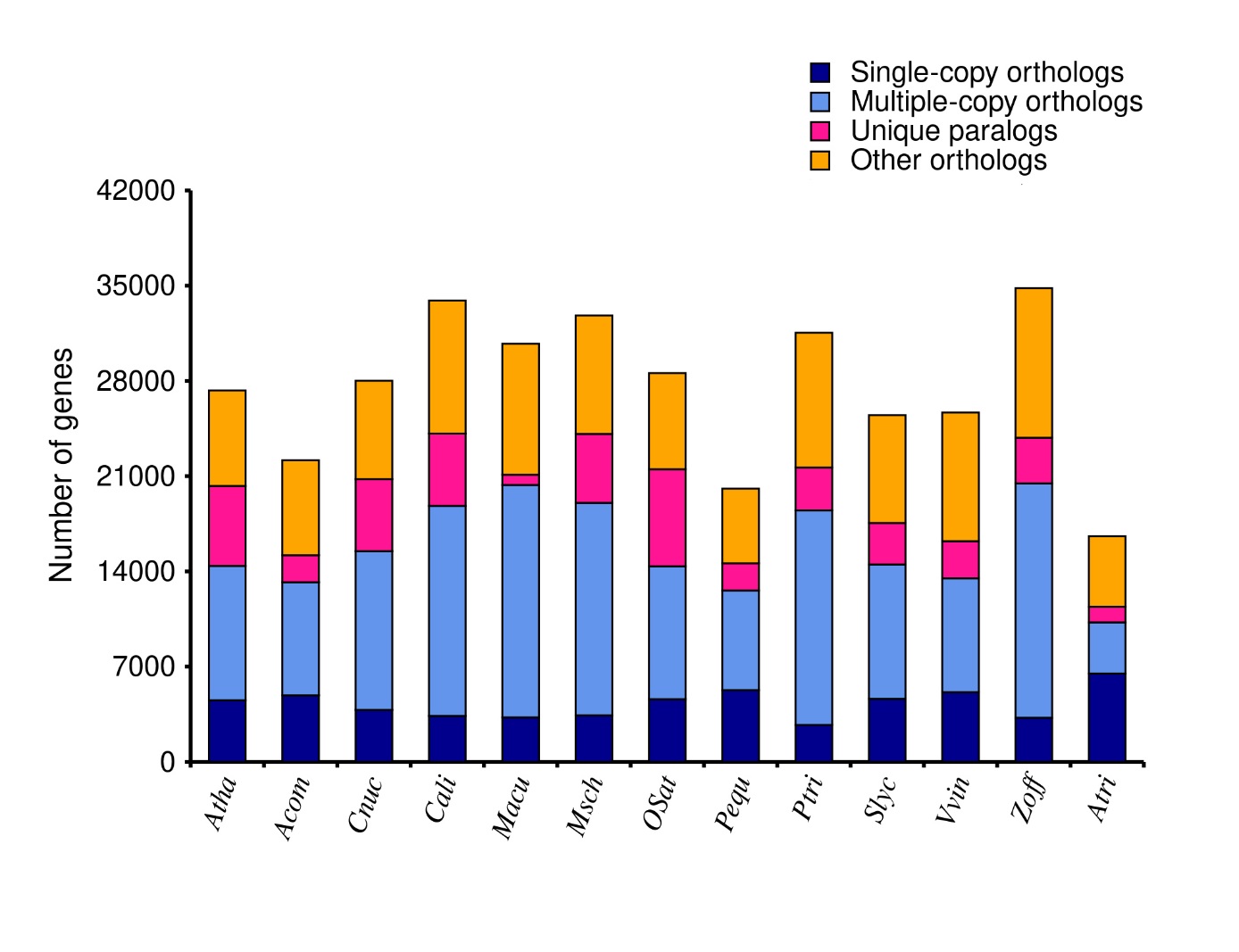
**

**Supplementary Figure 4.** Distribution of different types of orthologues in C. alismatifolia and other 12 plant species. “Unique paralogs” represents genes that only exist in one specific species, “Others orthologs” as unclassified orthologs.


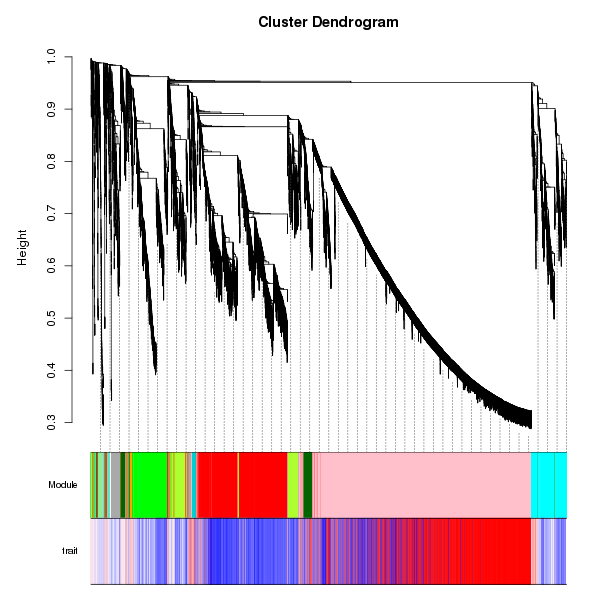


**Supplementary Figure5.** WGCNA coexpression network. Different modules are marked with different colors.

**
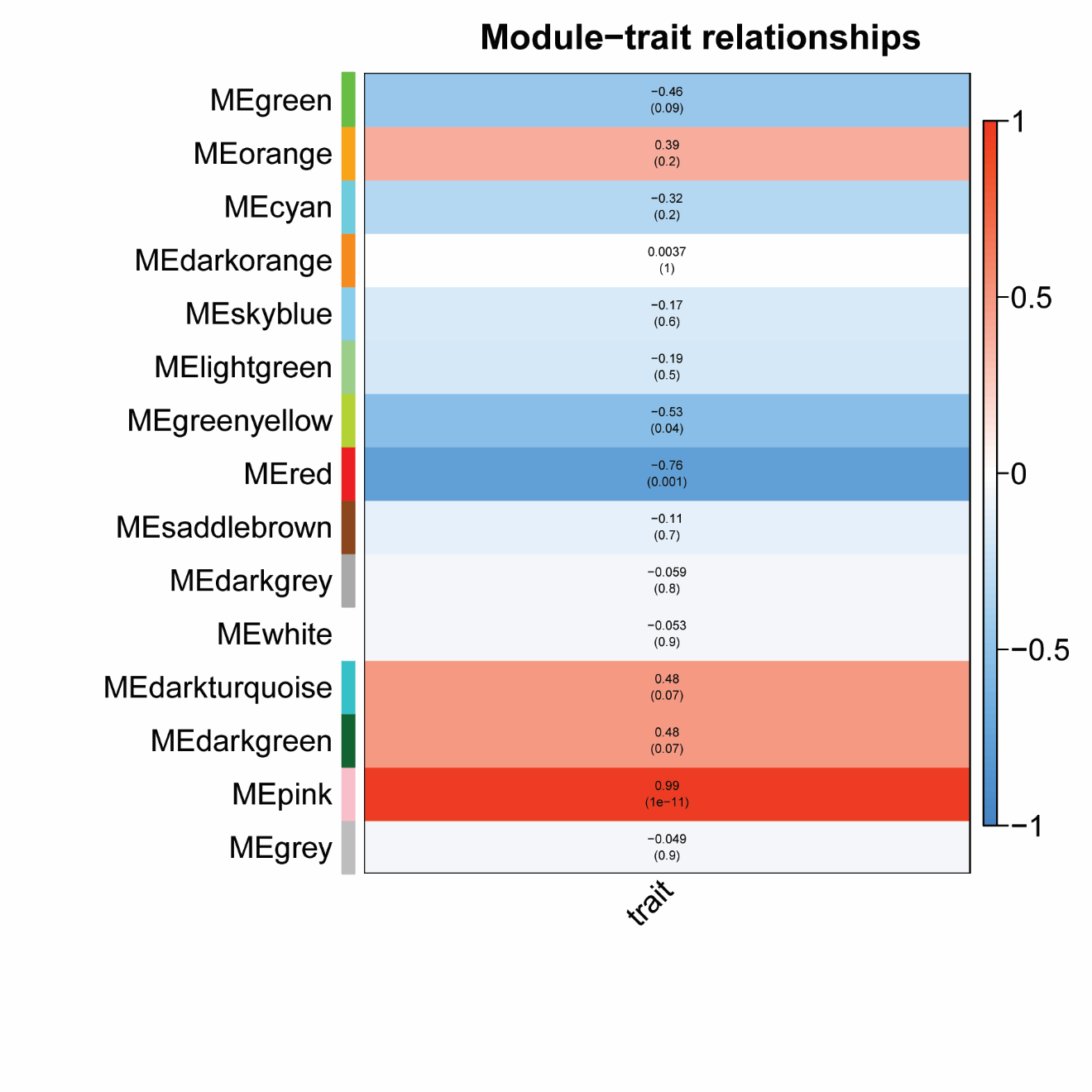
**

**Supplementary Figure 6.** Trait and module association analyses. Different colors designate the 14 different modules.

**
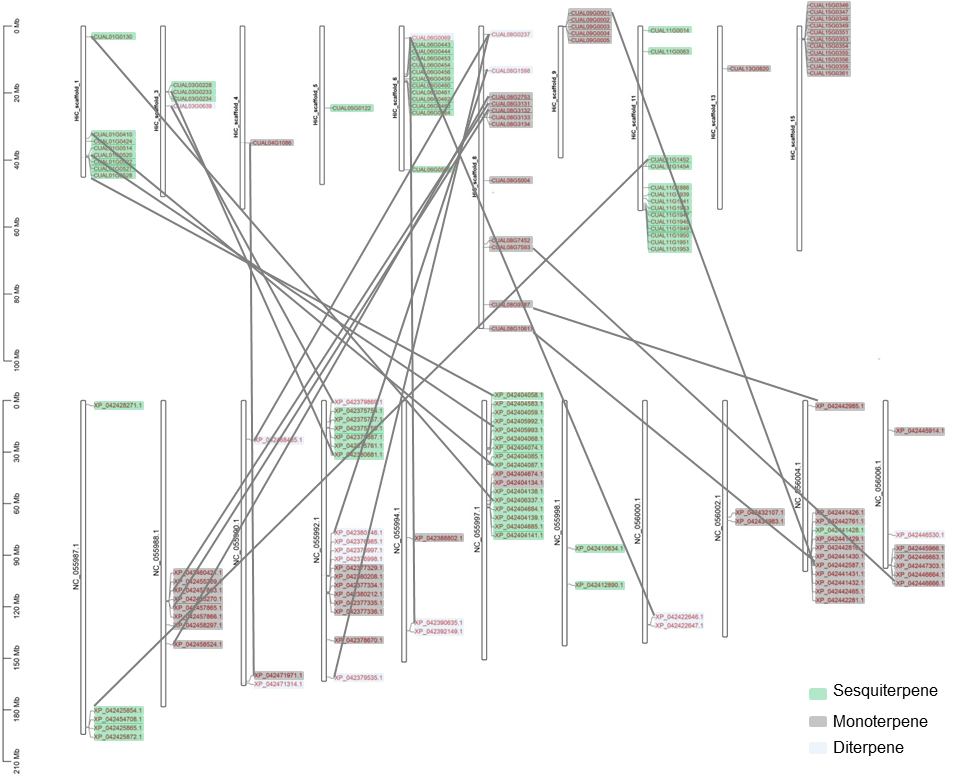
**

**Supplementary Figure 7.** Locations of terpene synthase genes in *C. alismatifolia* (upper) and *Z. officinale* (lower). Collinear gene pairs between the two species were marked using gray lines.

**
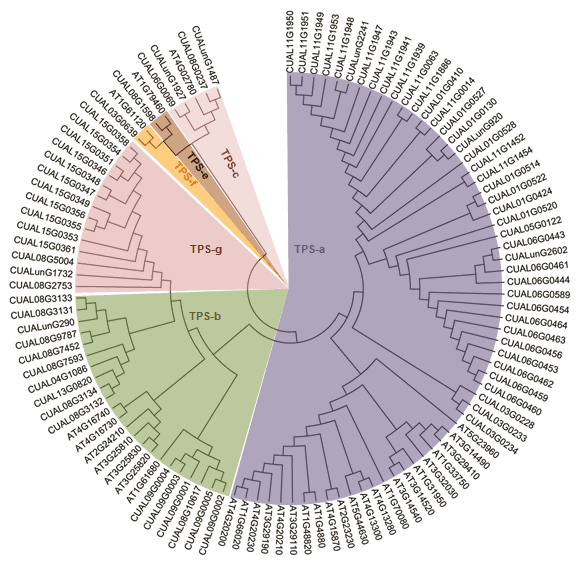
**

**Supplementary Figure 8.** The phylogenetic tree of terpene synthase genes in *C. alismatifolia* and *A. thaliana*.

**
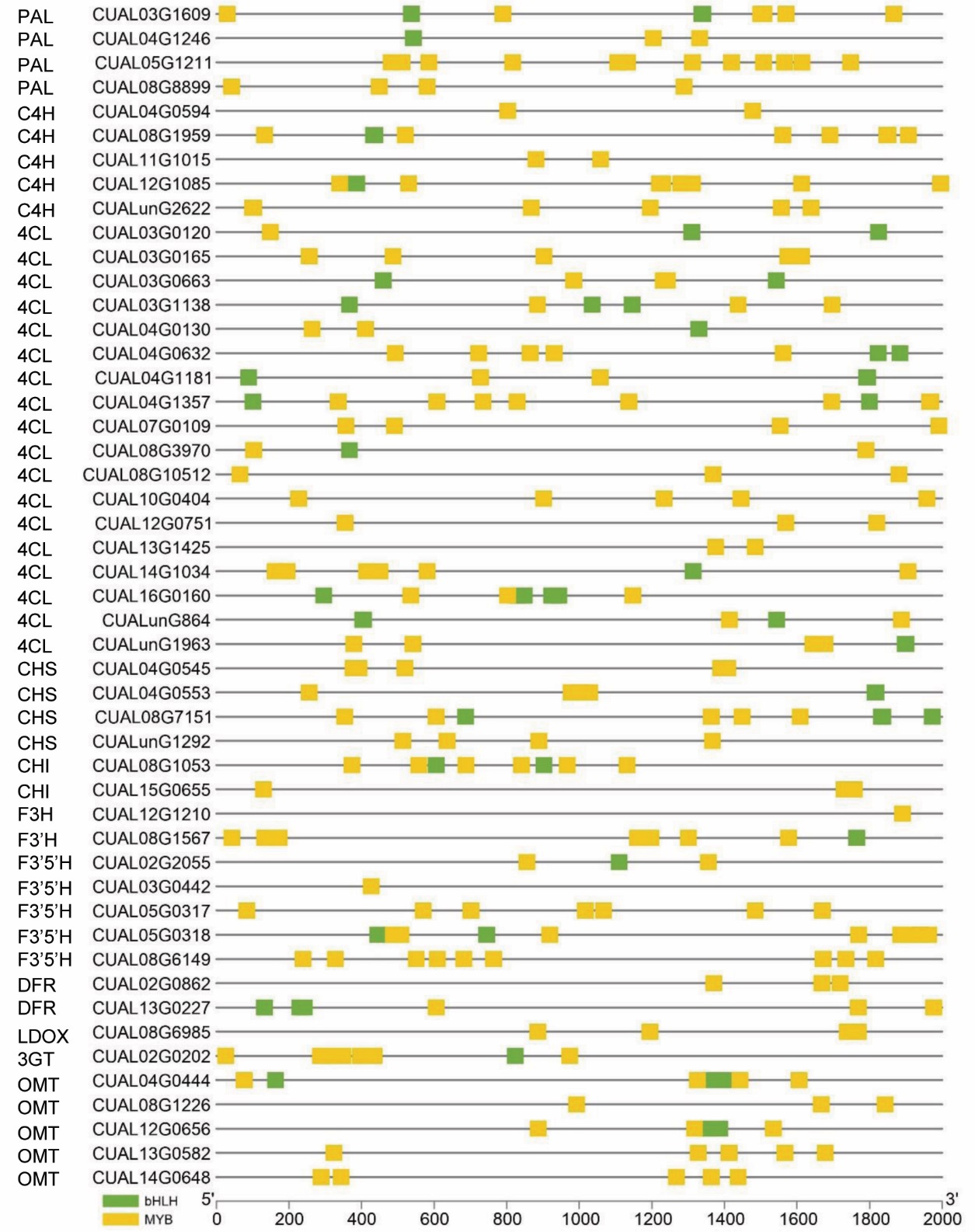
**

**Supplementary Figure 9.** Cis-acting regulatory elements in the promoter region of structural genes involved in anthocyanin biosynthesis.

**
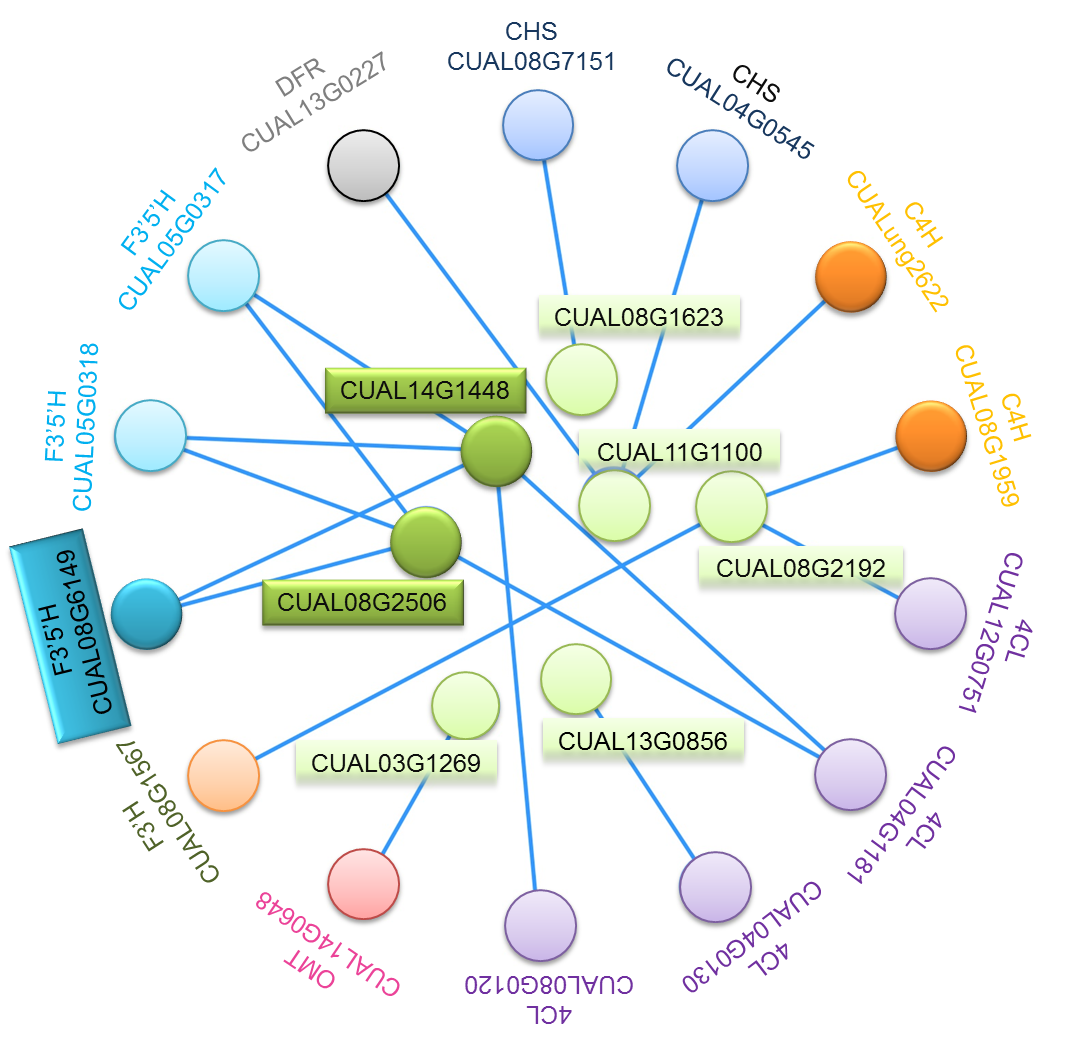
**

**Supplementary Figure 10** The co-expression network of seven R2R3-MYB transcription factors and 14 structural genes.

**
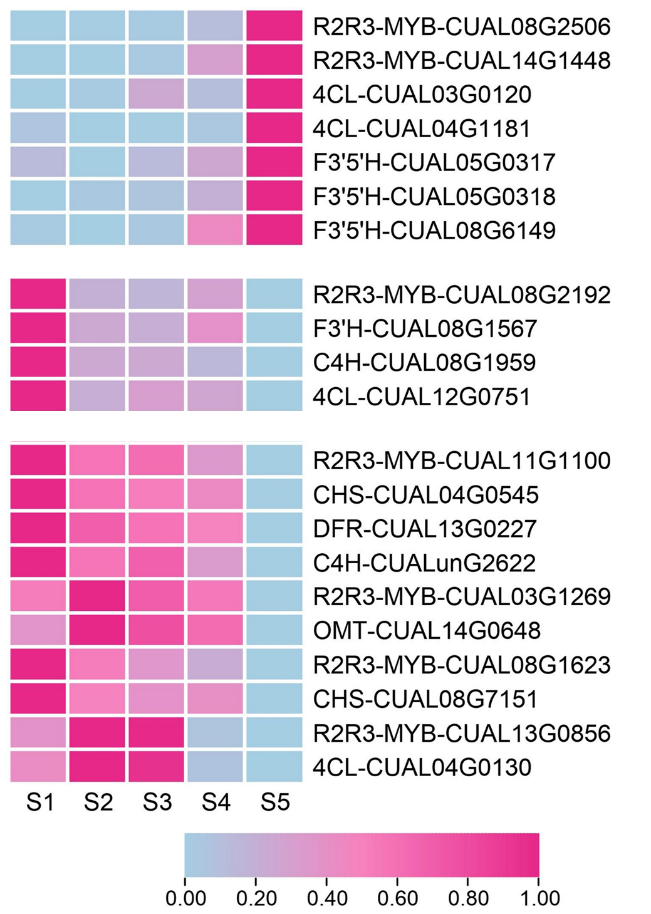
**

**Supplementary Figure 11.** Expression heatmap of seven R2R3-MYB genes and 14 structural genes. The expression values at the column scale were z-score normalized. Low to high expression is indicated by a change in color from blue to red.

**Supplementary Figure 12.** Expressions of two R2R3-MYB genes and the F3'5'H gene *CUAL08G6149*.


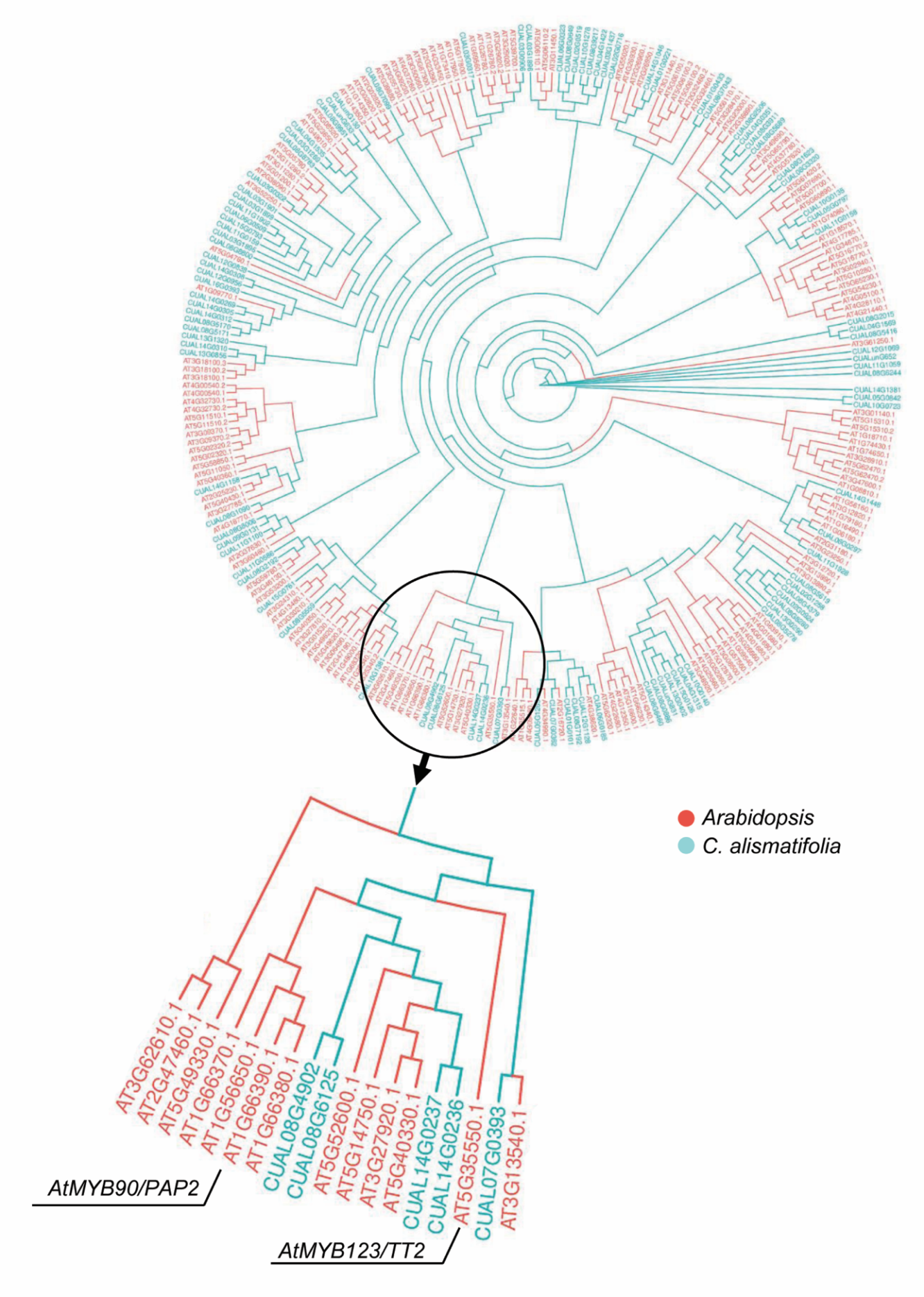


**Supplementary Figure 13.** The phylogenetic tree of *MYB* genes in *C. alismatifolia* and *A. thaliana*. The reported regulators of anthocyanin biosynthesis in *Arabidopsis* were underlined in black.


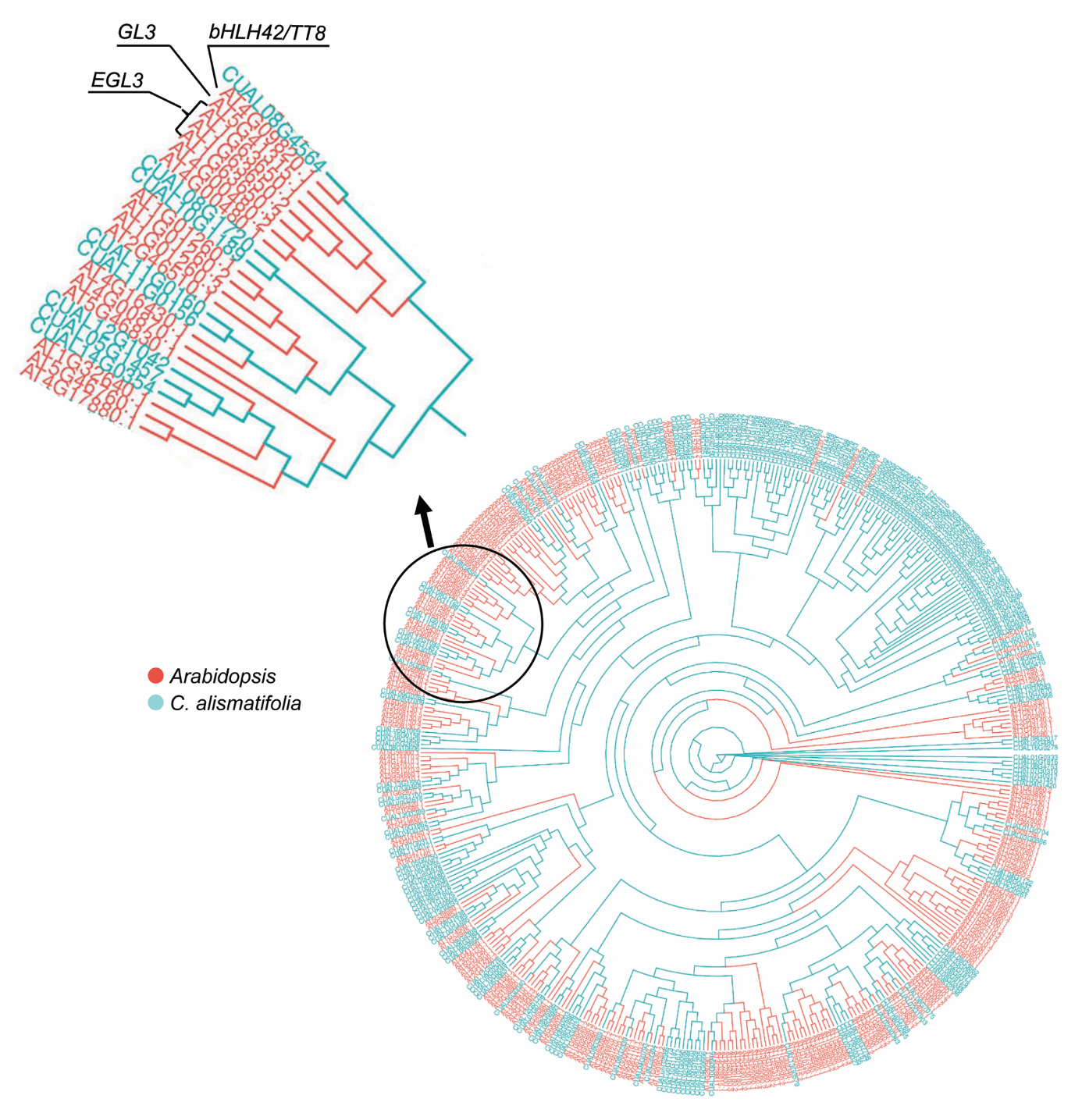


**Supplementary Figure 14.** The phylogenetic tree of *bHLH* genes in *C. alismatifolia* and *A. thaliana*. The reported regulators of anthocyanin biosynthesis in *Arabidopsis* were underlined in black.

**
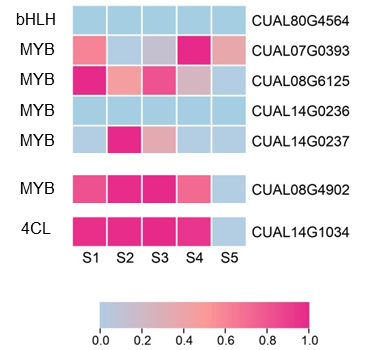
**

**Supplementary Figure 15.** Expression heatmap of six *C. alismatifolia* genes that were grouped into the same cluster with the reported regulators of anthocyanin biosynthesis in *Arabidopsis*. Among them, a MYB gene *CUAL08G2506* showed similar expression trends to the *4CL* gene *CUAL14G1034*. The expression values at the column scale were z-score normalized. Low to high expression is indicated by a change in color from blue to red.

**
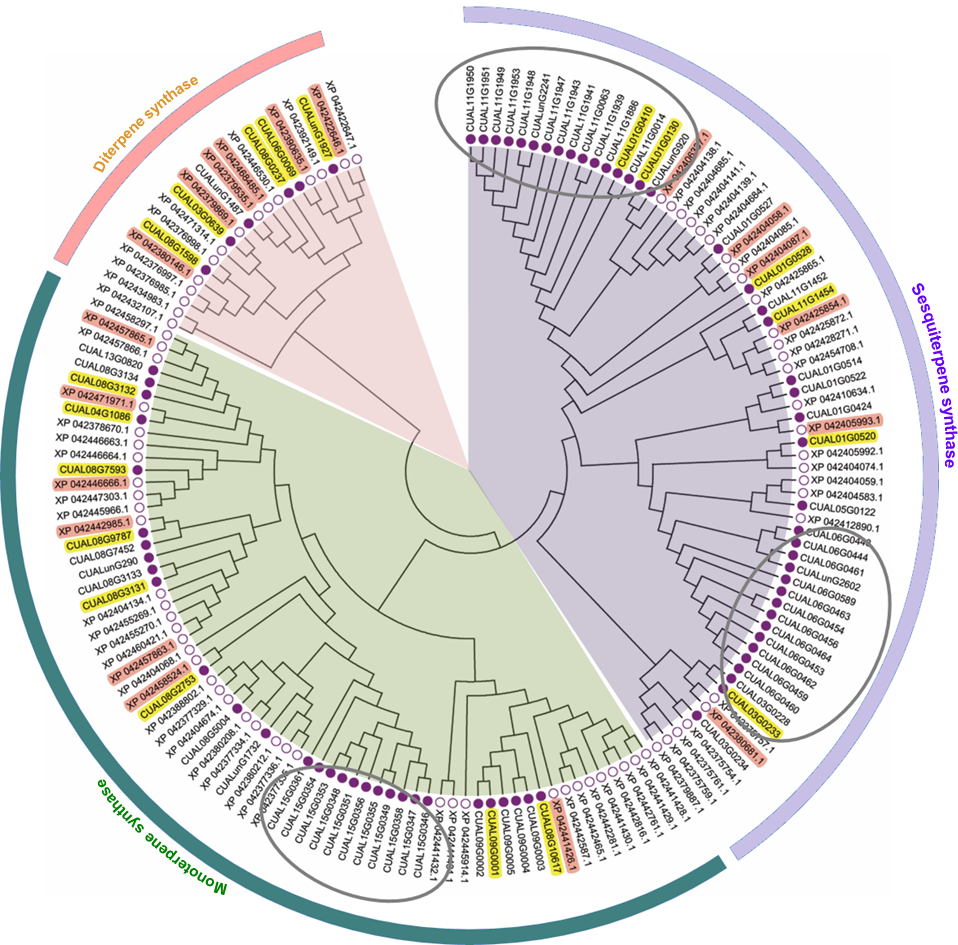
**

**Supplementary Figure 16.** The phylogenetic tree of terpene synthase genes in *C. alismatifolia* and *Z. officinale*. The orthologs in *C. alismatifolia* and *Z. officinale* are marked in yellow and red, respectively. Three large *C. alismatifolia* gene clusters that didn’t contain ortholog are marked in gray circles.

**
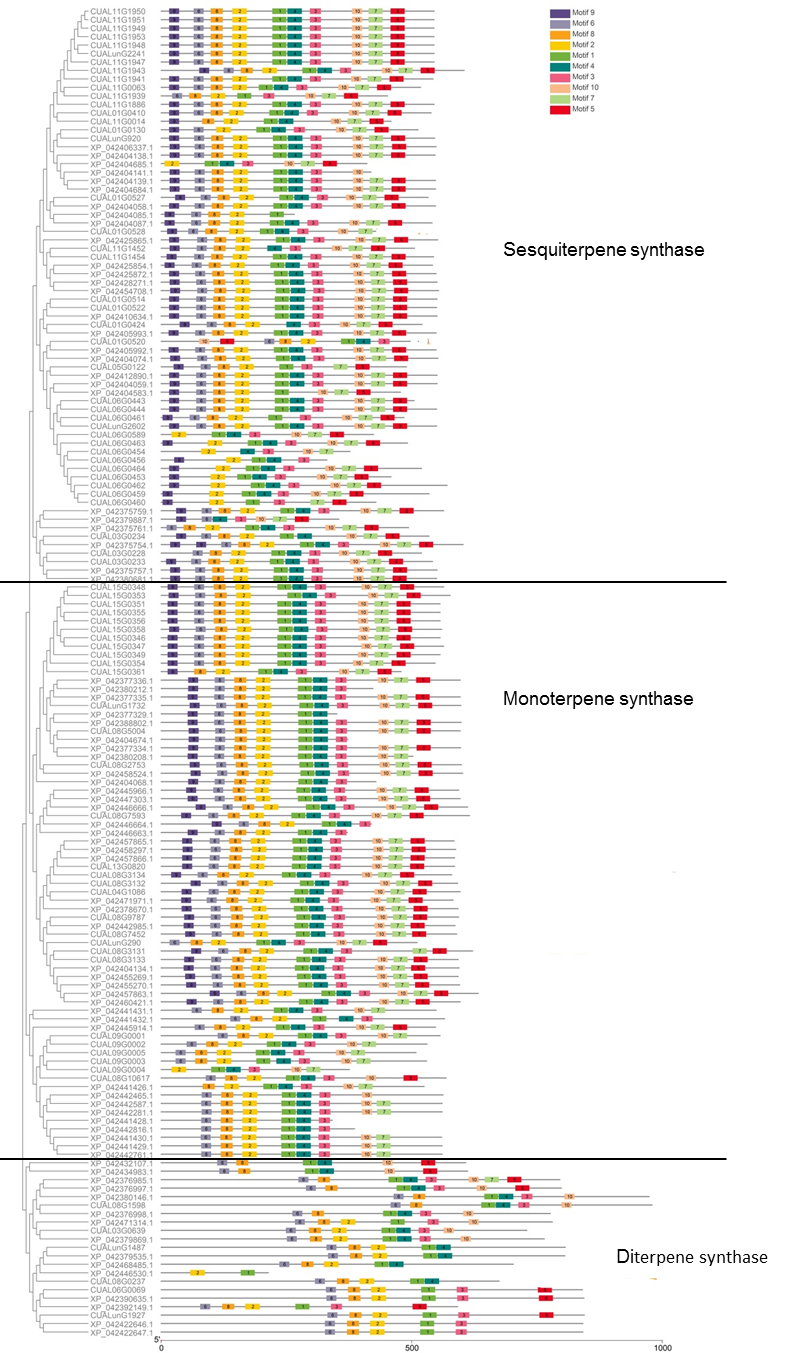
**

**Supplementary Figure 17.** The terpene synthase genes structure in *C. alismatifolia* and *Z. officinale*.

**Motif RR(X)8W DDXXD RXR**

CUAL04G1086 RRSANYQPNLW DDVYD RDR

CUAL08G2753 RRSANYQPNIW DDIYD RDR

CUAL08G3131 RKSTHYQPNFW DDVYD RDR

CUAL08G3132 RRSGNYKPNMW DDVYD RDR

CUAL08G3133 RKSTHYQPNFW DDVYD RDR

CUAL08G3134 RRSAHYQPNMW DDVYD RDR

CUAL08G5004 RRSGNYQPSIW DDIYD RDR

CUAL08G7452 RKSIKYQPNLW DDVYD RDR

CUAL08G7593 RRSGNYQPSIW DDVYD RDR

CUAL08G9787 RKYVQYPPNLR DDVYD RDR

CUAL09G0001 R---------- DDIFD RDQ

CUAL08G10617 ARPLQLQ-KQ- DDIFD RDQ

CUAL09G0002 --------LI- DDIFD RDQ

CUAL09G0003 -----MH---F DDIFD RDQ

CUAL09G0004 KELKEVT-SWW DDIFD RDQ

CUAL09G0005 ---MHFE-SL- DDIFD RDQ

CUAL13G0820 RRSAHYQPNMW DDVYD RDR

CUAL15G0346 RRSGNYHPNIW DDIYD RDR

CUAL15G0347 RRSGNYHPNIW DDIYD RDR

CUAL15G0348 RRSGNYHPNIW DDIYD RDR

CUAL15G0349 RRSGNYHPNIW DDIYD RDR

CUAL15G0351 RRSGNYHPNIW DDIYD RDR

CUAL15G0353 --VGELSPKIW DDIYD RDR

CUAL15G0354 RRSGNYHPNIW DDIYD RDR

CUAL15G0355 RRSGNYHPNIW DDIYD RDR

CUAL15G0356 RRSGNYHPNIW DDIYD RDR

CUAL15G0358 RRSGNYHPNIW DDIYD RDR

CUAL15G0361 RRSRNYHPNIW DDIYD RDR

CUALunG1732 RRSGNYHPNIW DDIYD RDR

CUALunG290 --------MTT DDVYD RDR

XP_042377329.1 RRSGNYQPSLW ----- RDR

XP_042377334.1 RRSGNYHPNIW DDIYD RDR

XP_042377335.1 RRSANYHPNIW DDIYD RDR

XP_042377336.1 RRSANYHPNIW DDIYD RDR

XP_042378670.1 RRSANYQPSLW DDVYD RDR

XP_042380208.1 RRSGNYHPSLW DDIYD RDR

XP_042380212.1 RRSANYHPNIW DDIYD RDR

XP_042388802.1 RRSGNYQPSIW DDIYD RDR

XP_042404134.1 GKSTHYRPNSW DDVYD RDR

XP_042404674.1 RRSGNYQPSIW DDIYD RDR

XP_042432107.1 -TVRTPPPSAI DDLMD RGG

XP_042434983.1 RPLSALSPAAA DDEMD RGG

XP_042441426.1 NPSSTHI---- DDIFD RDQ

XP_042441429.1 RLHQLQKQRIM DDIFD RDQ

XP_042441430.1 QLHQLQKQRIM DDIFD RDQ

XP_042441431.1 TKSPRHK---- DDIFD RDQ

XP_042441432.1 GGANYRRPCSW DDIFD RDQ

XP_042442281.1 RLHQLQKQRIM DDIFD RDQ

XP_042442465.1 RLHQLQKQRIM DDIFD RNQ

XP_042442587.1 RLHQLQKQRIM DDIFD RNQ

XP_042442761.1 RLHQLQKQRIM DDIFD RDQ

XP_042442816.1 RLHQLQKQRIM DDIFD RDQ

XP_042442985.1 RKYVQYPPNLR DDVYD RDR

XP_042445914.1 RKQQTTKP--- DDIFD RVQ

XP_042445966.1 RRSGNYQPSIW DDVYD RDR

XP_042446663.1 RRSGNYQPSIW DDVYD RDR

XP_042446664.1 RRSGNYQPSIW DDVYD RDR

XP_042446666.1 RRSGNYQPSIW DDVYD RDR

XP_042447303.1 RRSGNYQPSIW DDVYD RDR

XP_042455269.1 RKSTHYQPNSW DDVYD RDR

XP_042455270.1 RKSTHSKPNSW DDVYD RDS

XP_042457863.1 RSSTHYQPNSW DDVYD RDR

XP_042457865.1 RRSAHYQPNMW DDVYD RDR

XP_042457866.1 RRSAHYQPNMW DDVYD RDR

XP_042458297.1 RRSAHYQPNMW DDVYD RDR

XP_042458524.1 RRSANYQPNIW DDIYD RDR

XP_042460421.1 RSSTHYQPNSW DDVYD RDR

XP_042471971.1 RRSAIYQPNLW DDVYD RDR

**Supplementary Figure 18.** The conserved motif sequences of sesquiterpene synthase genes in *C. alismatifolia* and *Z. officinale*. The conserved amino acids are marked in color.

**Motif RR(X)8W DDXXD RXR**

CUAL01G0130 RNTTKYHPSIW DDIYD RDR

CUAL01G0410 RKTSKYHPSVW DDIYD RDR

CUAL01G0424 RKSTQYHPSVW DDIYD RDR

CUAL01G0514 RKSTKYHPSIW DDIYD RDR

CUAL01G0520 R---------- DDIYD RDR

CUAL01G0522 RKSTKYHPSIW DDIYD RDR

CUAL01G0527 HNTSKYHPSIW DDIYD RDR

CUAL01G0528 RNTSKYHPSIW DDIYD RDR

CUAL03G0228 -------P--W DDIYD RER

CUAL03G0233 RQLAGFDPSFW DDTYD RER

CUAL03G0234 RQLAGFDPSFW ----- RDR

CUAL05G0122 RKSAKYHPSVW DDIYD ---

CUAL06G0443 RKSSKFHPSIW DDIYD RDR

CUAL06G0444 RKSSKFHPSIW DDIYD RDR

CUAL06G0453 RKSSKFHPSIW VDIYD RDR

CUAL06G0454 -------M--- GDIYD RDR

CUAL06G0456 RKSSKFHPSIW DDIYD RDQ

CUAL06G0459 RKSSKFHPSIW DDIYD HDR

CUAL06G0460 HKSSKFHPSIW NDIYD RDL

CUAL06G0461 RKSSKFHPSIW DDIYD RDR

CUAL06G0462 RKLSKFHPRIW DDIYD RDQ

CUAL06G0463 RKSSKFHPSIW DDIYD RDR

CUAL06G0464 RKSSKFHPSIW DDIYD RDQ

CUAL06G0589 ----------- DDIYD RDR

CUAL11G0014 RKISKYHPSVW ----- RDR

CUAL11G0063 RKISKYHPSVW DDIYD RDR

CUAL11G1452 RKSAEYHPSVW DDIYD RDR

CUAL11G1454 RKSAEYHPSVW DDIYD RDR

CUAL11G1886 RKTSKYHPSVW DDIYD RDR

CUAL11G1939 ----------M DDIYD ---

CUAL11G1941 RKTSKYHPSVW DDIYD RDR

CUAL11G1943 RKTSKYHPSVW DDIYD RDR

CUAL11G1947 RKTSKYHPSVW DDIYD RDR

CUAL11G1948 RKTSKYHPSVW DDIYD RDR

CUAL11G1949 RKTSKYHPSVW DDIYD RDR

CUAL11G1950 RKTSKYHPSVW DDIYD RDR

CUAL11G1951 RKTSKYHPSVW DDIYD RDR

CUAL11G1953 RKTSKYHPSVW DDIYD RDR

CUALunG2241 RKTSKYHPSVW DDIYD RDR

CUALunG2602 RKSSKFHPSIW DDIYD RDR

CUALunG920 RKISKYHPCIW DDIYD RDR

XP_042375754.1 RQVAGFDPSFW DDTYD RDR

XP_042375757.1 RQVAGFDPSFW DDFYD RDR

XP_042375759.1 RQVAGFDLSFW DDIYD RER

XP_042375761.1 ----MFQ-NV- DDIYD RER

XP_042379887.1 RQVAGFDLSFC DDIYD RER

XP_042380681.1 RQVAGFDPSFW DDFYD RDR

XP_042404058.1 RKTSKYHPSVW DDIYD RDR

XP_042404059.1 RKSSKFHPSIW DDIYD RDR

XP_042404068.1 RRSGNYQPSLW DDIYD RDR

XP_042404074.1 RKSTKYHPSVW DDIYD RDR

XP_042404085.1 RKTSKYHSSVW ----- RDR

XP_042404087.1 RKTSKYHSSVW DDIYD RDR

XP_042404138.1 RKISKYHPSIW DDIYD RDR

XP_042404139.1 RKTSKYHPTIW DDIYD RDR

XP_042404141.1 RKTSKYHPTIW DDIYD RDR

XP_042404583.1 RKSSKFHPSIW ----- ---

XP_042404684.1 RKTSKYHPTI DDIYD RDR

XP_042404685.1 ---------- DDIYD RDR

XP_042405992.1 RKSSQYHPSVW DDIYD RDR

XP_042405993.1 HKSSKYHPSVW DDIYD RDR

XP_042406337.1 RKTSKYHPSIW DDIYD RDR

XP_042410634.1 RKSTKYHPSVW DDIYD RDR

XP_042412890.1 RKSTKFHPSIW DDIYD RDR

XP_042425854.1 RKSAEYHPTVW DDIYD RDR

XP_042425865.1 RKSAEYHPSVW DDIYD RDR

XP_042425872.1 RKSAEYHPSVW DDIYD RDR

XP_042428271.1 RKSAEYHPTVW DDIYD RDR

XP_042441428.1 RLHQLQKQRIM DDIFD RDQ

XP_042454708.1 RKSAEYHPTVW DDIYD RDR

**Supplementary Figure 19.** The conserved motif sequences of monoterpene synthase genes in *C. alismatifolia* and *Z. officinale*. The conserved amino acids are marked in color.
